# Increased Wnt and Notch signaling: a clue to the renal disease in Schimke immuno-osseous dysplasia?

**DOI:** 10.1186/s13023-016-0519-7

**Published:** 2016-11-05

**Authors:** Marie Morimoto, Clara Myung, Kimberly Beirnes, Kunho Choi, Yumi Asakura, Arend Bokenkamp, Dominique Bonneau, Milena Brugnara, Joel Charrow, Estelle Colin, Amira Davis, Georges Deschenes, Mattia Gentile, Mario Giordano, Andrew K. Gormley, Rajeshree Govender, Mark Joseph, Kory Keller, Evelyne Lerut, Elena Levtchenko, Laura Massella, Christy Mayfield, Behzad Najafian, David Parham, Jurgen Spranger, Peter Stenzel, Uluc Yis, Zhongxin Yu, Jonathan Zonana, Glenda Hendson, Cornelius F. Boerkoel

**Affiliations:** 1 Department of Medical Genetics, University of British Columbia, Vancouver, BC Canada; 2 Child & Family Research Institute, Vancouver, BC Canada; 3 Department of Endocrinology & Metabolism, Kanagawa Children’s Medical Center, Yokohama, Japan; 4 Department of Pediatric Nephrology, VU University Medical Center, Amsterdam, The Netherlands; 5 Département de Biochimie et Génétique, Centre Hospitalier Universitaire d’Angers, Angers, France; 6 Department of Pediatrics, University of Verona, Verona, Italy; 7 Division of Genetics, Birth Defects and Metabolism, Ann and Robert H. Lurie Children’s Hospital of Chicago, Northwestern University Feinberg School of Medicine, Chicago, IL USA; 8 Seattle Children’s Hospital, Seattle, WA USA; 9 Département de Pédiatrie, Hôpital Robert Debré, Paris, France; 10 Department of Medical Genetics, Hospital Di Venere – ASL Bari, Bari, Italy; 11 Pediatric Nephrology and Dialysis Unit, Ospedale Pediatrico Giovanni XXIII, Bari, Italy; 12 Department of Pediatrics, University of Oklahoma Health Sciences Center, Oklahoma City, OK USA; 13 Department of Pediatrics and Child Health, Nelson R. Mandela School of Medicine, University of KwaZulu-Natal, Durban, South Africa; 14 Department of Pediatric Nephrology, Phoenix Children’s Hospital, Phoenix, AZ USA; 15 Child Development and Rehabiliation Center, Oregon Institute on Disability & Development, Oregon Health & Science University, Portland, OR USA; 16 Department of Pathology, University Hospitals Leuven, Leuven, Belgium; 17 Department of Pediatric Nephrology, University Hospitals Leuven, Leuven, Belgium; 18 Division of Nephrology, Bambino Gesù Children’s Hospital and Research Institute, Rome, Italy; 19 Warren Clinic Family Medicine, Tulsa, OK USA; 20 Department of Pathology, University of Washington, Seattle, WA USA; 21 Department of Pathology, Children’s Hospital Los Angeles and Keck School of Medicine, University of Southern California, Los Angeles, CA USA; 22 Children’s Hospital, University of Mainz, Mainz, Germany; 23 Department of Pathology, Oregon Health and Science University, Portland, OR USA; 24 Department of Pediatrics, Division of Child Neurology, Dokuz Eylül University, School of Medicine, İzmir, Turkey; 25 Department of Pathology, University of Oklahoma Health Sciences Center, Oklahoma City, OK USA; 26 Department of Anatomic Pathology, Children’s and Women’s Health Centre of British Columbia, Vancouver, BC Canada; 27 Provincial Medical Genetics Program, Department of Medical Genetics, Children’s and Women’s Health Centre of British Columbia, 4500 Oak Street, Room C234, Vancouver, BC V6H 3N1 Canada

**Keywords:** Schimke immuno-osseous dysplasia, SMARCAL1 protein, Focal segmental glomerulosclerosis, Wnt signaling pathway, Notch signaling pathway

## Abstract

**Background:**

Schimke immuno-osseous dysplasia (SIOD) is a multisystemic disorder caused by biallelic mutations in the SWI/SNF-related matrix-associated actin-dependent regulator of chromatin, subfamily A-like 1 (*SMARCAL1*) gene. Changes in gene expression underlie the arteriosclerosis and T-cell immunodeficiency of SIOD; therefore, we hypothesized that SMARCAL1 deficiency causes the focal segmental glomerulosclerosis (FSGS) of SIOD by altering renal gene expression. We tested this hypothesis by gene expression analysis of an SIOD patient kidney and verified these findings through immunofluorescent analysis in additional SIOD patients and a genetic interaction analysis in *Drosophila*.

**Results:**

We found increased expression of components and targets of the Wnt and Notch signaling pathways in the SIOD patient kidney, increased levels of unphosphorylated β-catenin and Notch1 intracellular domain in the glomeruli of most SIOD patient kidneys, and genetic interaction between the *Drosophila SMARCAL1* homologue *Marcal1* and genes of the Wnt and Notch signaling pathways.

**Conclusions:**

We conclude that increased Wnt and Notch activity result from SMARCAL1 deficiency and, as established causes of FSGS, contribute to the renal disease of most SIOD patients. This further clarifies the pathogenesis of SIOD and will hopefully direct potential therapeutic approaches for SIOD patients.

**Electronic supplementary material:**

The online version of this article (doi:10.1186/s13023-016-0519-7) contains supplementary material, which is available to authorized users.

## Background

Schimke immuno-osseous dysplasia (SIOD, OMIM 242900) is an autosomal recessive disease; its prominent features are facial dysmorphism, hyperpigmented macules, focal segmental glomerulosclerosis (FSGS), spondyloepiphyseal dysplasia, and T-cell immunodeficiency [1–3]. Additional features include hypothyroidism, abnormal dentition, bone marrow failure, thin hair, corneal opacities, arteriosclerosis, cerebral ischemia, and migraine-like headaches [2–5].

The renal disease begins as proteinuria, progresses to steroid-resistant nephropathy, and ultimately advances to end-stage renal disease [4, 6]. FSGS is the predominant renal pathology and is refractory to treatment with glucocorticoids, cyclosporine A, and cyclophosphamide [4, 6]. Suggesting a cell autonomous mechanism for the renal disease, renal transplantation is efficacious, and the disease does not recur in the graft [2, 4, 5].

Biallelic mutations of the SWI/SNF-related matrix-associated actin-dependent regulator of chromatin, subfamily A-like 1 (*SMARCAL1*) gene cause SIOD [7]. *SMARCAL1* encodes a DNA annealing helicase that is a distant member of the SWI/SNF family of ATP-dependent chromatin remodeling proteins [8]. SMARCAL1 recognizes DNA structure, binds to open chromatin, is involved in the DNA damage response [9, 10] and DNA replication fork restart [11, 12], and, along with genetic and environmental factors, alters gene expression [13].

Gene expression changes appear critical to SIOD pathology. Full or partial explanations for the vascular disease and T-cell immunodeficiency of SIOD patients are respectively decreased expression of elastin (*ELN*) in the aorta [14–16] and of interleukin 7 receptor alpha chain (*IL7R*) in the T cells [17–19].

Based on these findings, we hypothesized that SMARCAL1 deficiency causes the renal disease of SIOD by altering gene expression. Studies of other glomerulopathies find increased Wnt [20–23] and Notch signaling [24–27] as causes of podocyte dysfunction. Canonical Wnt pathway activation proceeds via inhibition of β-catenin ubiquitination, saturation of the β-catenin destruction complex, cytoplasmic accumulation and nuclear translocation of newly synthesized unphosphorylated β-catenin, and subsequent activation of target gene transcription through interaction with transcription factors and transcriptional co-activators [28]. Notch pathway activation involves proteolytic cleavage of the Notch transmembrane receptor by an ADAM metalloproteinase and the γ-secretase complex, nuclear translocation of the released Notch1 intracellular domain (NICD), and subsequent activation of target gene transcription through interaction of the NICD with transcription factors and transcriptional co-activators [29]. Wnt and Notch signaling are critical for kidney development and become undetectable in the glomeruli of the postnatal kidney [26, 30].

Analyses presented herein showed upregulation of the Wnt and Notch signaling pathways in the SIOD kidney and genetic interaction between the *Drosophila SMARCAL1* homologue and genes encoding components of the Wnt and Notch pathways. We suggest therefore that the upregulation of the Wnt and/or Notch pathways contributes to the renal disease in SIOD.

## Methods

### Patients and human tissues

The guardians of the patients referred to this study signed informed consent approved by the Research Ethics Board of the University of British Columbia (Vancouver, BC, Canada). Autopsy and biopsy tissues were obtained according to the protocol approved by the University of British Columbia (Vancouver, BC, Canada). The renal parameters and the *SMARCAL1* mutations of the SIOD patients included in the study are listed in Table 1 and Additional file 1: Table S1, respectively.

**Table 1:** The renal parameters of the SIOD patients included in this study

Patient ID	Age at onset (years)	Age at death (years)	Nephrotic syndrome	Hypertension	Proteinuria	Hypercholesterolemia	Renal dialysis	Age at renal dialysis (years)	Renal transplant	Age at renal transplantation (years)	Renal pathology
SD4b	3	8	+	+	+	?	−	n/a	−	n/a	FSGS
SD26	<4	8	+	+	+	+	+	5	−	n/a	FSGS
SD60	7	13.7	+	+	+	?	+	12.5	+	13	FSGS
SD79	<4	10	+	−	+	−	−	n/a	−	n/a	FSGS
SD120	4.5	5.4	+	+	+	+	−	n/a	−	n/a	FSGS
SD121	2.5	4.8	+	−	+	+	−	n/a	−	n/a	Diffuse podocytopathy with early features of FSGS
SD131	3	4.6	+	+	+	+	+	3.8	−	n/a	Global glomerulosclerosis likely secondary to FSGS
SD146	2	4	−	−	+	+	−	n/a	−	n/a	FSGS

*Abbreviations*: *+* present, *−* absent, *?* unknown, *FSGS* focal segmental glomerulosclerosis, *ID* identification, *n/a* not applicable, *SIOD* Schimke immuno-osseous dysplasia

In accordance with institutional policies as approved by the Institutional Review Board (41557) at the University of Washington, human fetal kidney from second trimester elective terminations were provided as de-identified specimens by the Laboratory of Developmental Biology at the University of Washington (Seattle, WA), a National Institute of Child Health & Human Development supported program. De-identified control specimens provided according to the protocol H06-70283 approved by the Clinical Research Ethics Board at the University of British Columbia (Vancouver, BC, Canada) included renal biopsy sections from ten pediatric patients with isolated FSGS, postmortem kidney tissue from four pediatric patients, a skin biopsy from a 16-year-old female, and adenoma tissue from a 17-year-old female with familial adenomatous polyposis. Sample characteristics and use are summarized in Additional file 1: Table S2.

### *Drosophila melanogaster* lines

The loss-of-function mutant *Marcal1* ^*del*^ and the *Marcal1* overexpression transgenic line *pUAST-Marcal1/CyO*; *tubulin-GAL4/TM3, Sb* ^*1*^ have been previously described [13] (Additional file 1: Figure S1). The *C96-GAL4 UAS-Hrs/MKRS* transgenic line, used to control for non-specific interactions with the GAL4-UAS system, was a gift from Dr. Hugo Bellen (Baylor College of Medicine, Houston, TX, USA). All other *Drosophila* stocks were obtained from the Bloomington *Drosophila* Stock Center (Bloomington, IN, USA).

### RNA extraction

Total RNA was extracted from flash frozen kidney pulverized with a Bessman tissue pulverizer (Spectrum Laboratories, Rancho Dominguez, CA, USA) or from 8 *Drosophila* adult female flies of each genotype by using the RNeasy Mini Kit (Qiagen, Toronto, ON, Canada). Total RNA from formalin-fixed paraffin-embedded (FFPE) fetal kidney was isolated using the RNeasy FFPE Kit (Qiagen, Toronto, ON, Canada). Genomic DNA was removed by on-column DNase I digestion (Qiagen, Toronto, ON, Canada).

### RNA-seq and KEGG pathway analysis

Strand-specific, paired-end RNA-seq on poly(A) RNA was performed by Macrogen (Seoul, Korea) using the TruSeq Stranded Total RNA Library Prep Kit (Illumina, San Diego, CA) and the HiSeq 2000 System (Illumina, San Diego, CA). This kit depleted the ribosomal RNA (rRNA) using Ribo-Zero rRNA reduction chemistry. Quantification was performed by calculating fragments per kilobase per million mapped reads (FPKM). Prior to fold change calculation and log_2_ transformation, a pseudocount of 1 was added to each FPKM value to reduce the inherent bias of finding gene expression changes in those genes where one sample has very little or no detectable gene expression [31]. The threshold for differential gene expression between the kidney from the SIOD patient and sex-matched unaffected control was set at log_2_ fold change (i.e., log_2_ (FPKM_SIOD_ + 1/FPKM_UNAFFECTED_ + 1)) > 1 or < −1. The Kyoto Encyclopedia of Genes and Genomes (KEGG) pathway analysis was performed with the online bioinformatic resource Database for Annotation, Visualization, and Integrated Discovery (DAVID) version 6.7 available at https://david.ncifcrf.gov.

### Reverse transcription

For total RNA extracted from flash frozen kidney, reverse transcription was performed with the RT^2^ First Strand Kit (Qiagen, Toronto, ON, Canada). For total RNA extracted from FFPE kidney or adult flies, reverse transcription was performed with the qScript cDNA SuperMix (Quanta Biosciences, Gaithersburg, MD, USA).

### Gene expression arrays

The Wnt (PAHS-043Y) and Notch (PAHS-059Y) Signaling Pathway Plus PCR Arrays (Qiagen, Toronto, ON, Canada) and the RT^2^ Real-Time SYBR Green/Rox PCR Master Mix (Qiagen, Toronto, ON, Canada) were used to assess mRNA levels between the sex-matched unaffected control and the SIOD kidney according to the manufacturer’s specifications. The threshold for calling differential mRNA levels was a log_2_ fold change > 1 or < −1 and a *p* value of less than 0.05.

### Quantitative PCR

SsoFast EvaGreen Supermix (Bio-Rad Laboratories, Mississauga, ON, Canada) was used with the StepOnePlus Real-Time PCR System (Applied Biosystems, Thermo Fisher Scientific, Waltham, MA, USA) for quantitative PCR. Human *GAPDH* and *Drosophila Gapdh2* housekeeping genes were used as endogenous controls. The primer sequences used in this study are listed in Additional file 1: Table S3.

### Indirect immunofluorescence

FFPE sections of tissue or cell pellets were cut at 5 microns. Following deparaffinization and rehydration, heat induced epitope retrieval was performed with sodium citrate buffer (10 mM sodium citrate, 0.05 % Tween 20, pH 6.0). Endogenous peroxidases were inactivated for 1 h at room temperature by incubating the sections with peroxidase quenching buffer (3 % hydrogen peroxide in 1× phosphate-buffered saline (PBS), 0.1 % Tween 20, pH 7.4 (PBSTw) for unphosphorylated β-catenin immunofluorescent staining or 1× PBS, 0.2 % Triton X-100, pH 7.4 (PBST) for the Notch1 intracellular domain (NICD) immunofluorescent staining). Non-specific protein binding was blocked by incubating the sections with blocking buffer (20 % normal goat serum, 10 % bovine serum albumin, 1× casein (Vector Laboratories, Burlington, ON, Canada) in PBSTw or PBST) overnight at 4 °C. Endogenous biotin, biotin receptors, and avidin binding sites were blocked with the Avidin/Biotin Blocking Kit (Vector Laboratories, Burlington, ON, Canada).

Rabbit anti-unphosphorylated β-catenin (clone D13A1, Cell Signaling Technology, Danvers, MA, USA) or rabbit anti-NICD (ab8925, Abcam, Toronto, ON, Canada) were used as primary antibodies. A biotinylated anti-rabbit IgG secondary antibody was used to detect the primary antibodies. Horseradish peroxidase-conjugated streptavidin was then used to detect the biotinylated anti-rabbit IgG secondary antibody. Subsequently, tyramide labeling was performed using Alexa Fluor 594 tyramide (Invitrogen, Thermo Fisher Scientific, Waltham, MA, USA). ProLong Gold Antifade Mountant with 4′, 6-diamidino-2-phenylindole (DAPI) (Invitrogen, Thermo Fisher Scientific, Waltham, MA, USA) was used to mount the sections and counterstain the DNA. Representative images were acquired using a 20×/0.75 Plan-APOCHROMAT, 40×/1.3 oil DIC Plan-NEOFLUAR, or 100×/1.30 oil Plan-NEOFLUAR objective lens on an Axiovert 200 inverted microscope, an AxioCam MR microscope camera, and the AxioVision software version 4.8 (Carl Zeiss, Toronto, ON, Canada). The glomerular β-catenin signal was quantified for each sample (see Additional file 1: Methods for further details).

### *Drosophila* genetics studies

We performed an overexpression and loss-of-function genetic screen in *Drosophila* to determine whether the *SMARCAL1* homologue *Marcal1* genetically interacts with Wnt and Notch pathway genes (see Additional file 1: Methods for further details).

### Statistics

For the KEGG pathway analysis, enrichment *p* values were corrected for multiple comparisons by the Bonferroni method. A *p* value of less than 0.05 was considered statistically significant. For the PCR expression arrays, data were analyzed by the 2-tailed Student’s *t*-test. A *p* value of less than 0.05 was considered statistically significant.

## Results

### Genome-wide gene expression analysis identifies increased mRNA levels of Wnt signaling pathway and target genes in an SIOD patient kidney

**Fig. 1:**
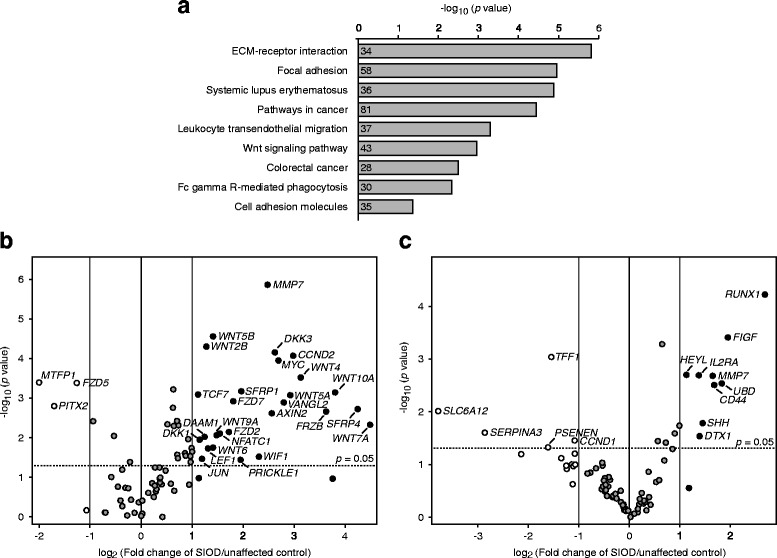
Genome-wide and targeted gene expression analyses in an SIOD patient kidney. **a** Kyoto Encyclopedia of Genes and Genomes (KEGG) pathway enrichment analysis of upregulated genes (log_2_ fold change > 1) in an SIOD kidney compared to a sex-matched unaffected control kidney. A Bonferroni-corrected *p* value of < 0.05 was used as a threshold for determining significant KEGG pathways. The horizontal axis represents the -log_10_ (*p* value) of significant KEGG pathways. The number of unique DAVID gene IDs involved in a given term is indicated within the bar representing each pathway. **b** and **c** Volcano plots comparing the expression of Wnt (**b**) and Notch (**c**) pathway genes and targets in an SIOD patient kidney to an unaffected control kidney. White, grey, and black dots respectively represent downregulated (log_2_ fold change < −1), unchanged, and upregulated (log_2_ fold change > 1) expression in the SIOD kidney versus the unaffected control kidney. For genes above the dotted line, the differential expression has a *p* value of less than 0.05. Abbreviations: ECM, extracellular matrix; SIOD, Schimke immuno-osseous dysplasia

We hypothesized that SMARCAL1 deficiency leads to gene expression changes that contribute to the pathogenesis of the renal disease in SIOD. To test this, we used RNA-seq to compare the transcriptomes of kidney tissue from a 5.4-year-old male SIOD patient and a 3-year-old unaffected male. This comparison detected 2241 genes with increased mRNA levels (log_2_ fold change > 1) and 892 genes with decreased mRNA levels (log_2_ fold change < −1) in the SIOD kidney tissue. After Bonferroni correction, KEGG pathway analysis of the genes with decreased mRNA levels did not reveal any significantly enriched pathways. In contrast, KEGG pathway analysis of genes with increased mRNA levels revealed significantly enriched pathways of cellular adhesion (e.g., focal adhesion, cell adhesion molecules), immune function (e.g., leukocyte transendothelial migration, Fc gamma R-mediated phagocytosis), disease (e.g., systemic lupus erythematosus, pathways in cancer, colorectal cancer), and Wnt signaling (Fig. 1a and Additional file 1: Table S4).

### Targeted gene expression analysis detects increased mRNA levels of Wnt and Notch signaling pathway and target genes in an SIOD patient kidney

Given that upregulation of the Wnt pathway [20–23] or the Notch pathway [24–27] is a cause of glomerulopathy, we measured mRNA levels of Wnt and Notch signaling pathway and target genes using the RT^2^ Profiler PCR Arrays. These analyses showed that of the 84 Wnt pathway-related genes tested, 30 were differentially expressed (Fig. 1b and Additional file 1: Table S5) and that of the 84 Notch pathway-related genes tested, 14 were differentially expressed (Fig. 1c and Additional file 1: Table S6). Wnt pathway-related genes with increased mRNA levels included ligands (e.g., *WNT2B*, *WNT4*, *WNT6*, *WNT7A*, *WNT10A*), components (e.g., *AXIN2*, *FZD2*, *FZD7*, *SFRP1*, *SFRP4*), and targets (e.g., *AXIN2*, *CCND2*, *JUN*, *MMP7*, *MYC*). Notch pathway-related genes with increased mRNA levels included components (e.g., *DTX1*) and targets (e.g., *HEYL*, *IL2RA*).

### Markers of Wnt and Notch pathway activation are increased in the glomerular cells of postnatal SIOD patient kidneys comparable to isolated FSGS controls

**Fig. 2:**
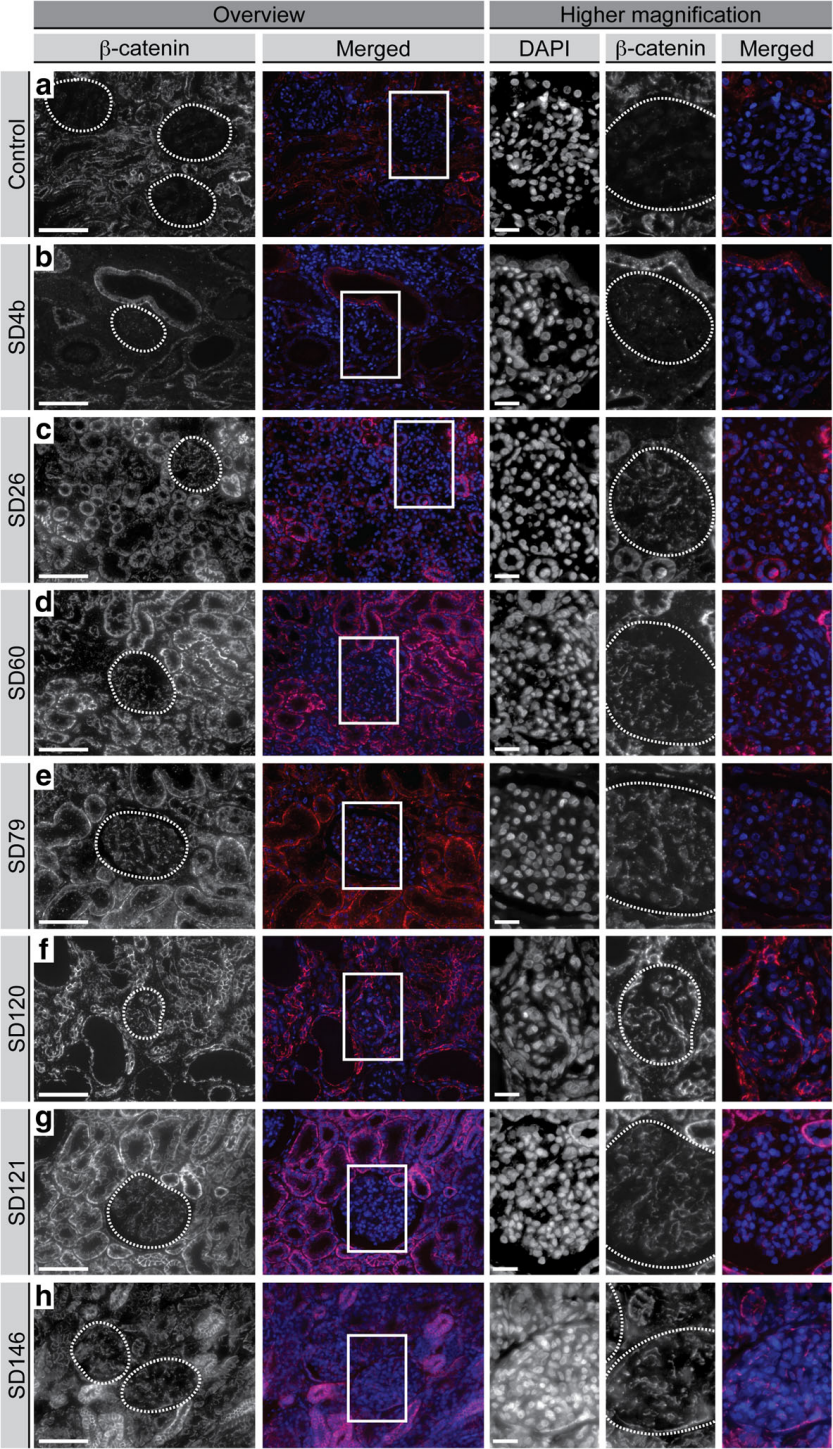
Immunofluorescent detection of unphosphorylated β-catenin in the glomerular cells of SIOD patient and unaffected control kidneys. Immunostaining with anti-unphosphorylated β-catenin (Alexa Fluor 594) in unaffected control kidney (**a**) and SIOD patient kidneys (**b**-**h**). The nuclei were counterstained with 4', 6-diamidino-2-phenylindole (DAPI). The boxed regions correspond to the higher magnification images on the right. The glomeruli have been outlined to aid in the visualization of β-catenin expression. Scale bars: overview images (200×) and higher magnification images (400×) = 100 microns. Abbreviations: DAPI, 4', 6-diamidino-2-phenylindole

**Fig. 3:**
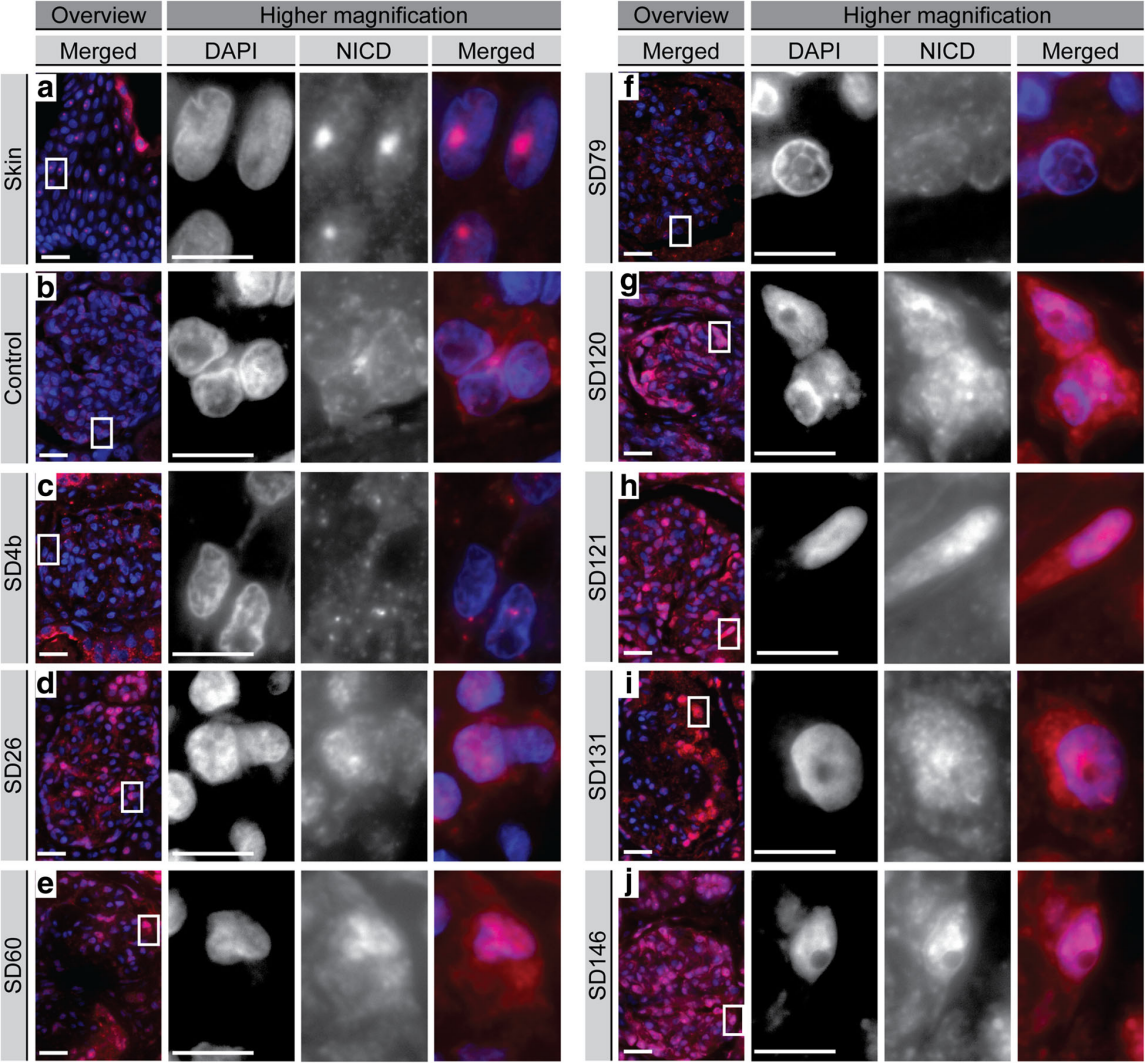
Immunofluorescent detection of the Notch1 intracellular domain (NICD) in the glomerular cells of SIOD patient and unaffected control kidneys. Immunostaining with anti-NICD (Alexa Fluor 594) in positive control skin (**a**), unaffected control kidney (**b**), and SIOD patient kidneys (**c**-**j**). The nuclei were counterstained with 4', 6-diamidino-2-phenylindole (DAPI). The boxed regions on the left correspond to the higher magnification images on the right. Scale bars: overview images (400×) = 100 microns; higher magnification images (1000×) = 10 microns. Abbreviations: DAPI, 4', 6-diamidino-2-phenylindole; NICD, Notch1 intracellular domain

Having established that several Wnt and Notch pathway-related genes and targets have altered expression in an SIOD kidney, we hypothesized that increased Wnt and Notch pathway signaling within the glomeruli contributes to the pathogenesis of FSGS in SIOD. To test this in additional SIOD patients, we used indirect immunofluorescence to profile the expression of unphosphorylated β-catenin and the nuclear localization of the Notch1 intracellular domain (NICD), which are respectively markers of canonical Wnt and Notch pathway activation [28, 29] (Additional file 1: Figure S2 and Fig. 3a). Compared to unaffected controls, most SIOD samples had increased glomerular staining for unphosphorylated β-catenin (6 of 7 patients) and nuclear NICD (6 of 8 patients) (Fig. 2, Fig. 3, Table 2, and Additional file 1: Figure S3). Similarly, most isolated FSGS samples had increased glomerular staining for unphosphorylated β-catenin (8 of 9 patients) and nuclear NICD (8 of 9 patients (Additional file 1: Figure S3, Figure S4, Figure S5, and Table 2).

**Table 2:** Summary of the β-catenin and NICD immunofluorescent analyses in SIOD and isolated FSGS patient kidney tissue

Patient ID	Unphosphorylated β-catenin expression	Nuclear NICD expression
SIOD patients		
SD4b	=	=
SD26	**↑**	**↑**
SD60	**↑**	**↑**
SD60 Tx	=	=
SD79	**↑**	=
SD120	**↑**	**↑**
SD121	**↑**	**↑**
SD131	n/a^a^	**↑**
SD146	**↑**	**↑**
Isolated FSGS patients		
FSGS-1	**↑**	=
FSGS-2	**↑**	**↑**
FSGS-3	**↑**	**↑**
FSGS-4	**↑**	**↑**
FSGS-5	**↑**	**↑**
FSGS-6	**↑**	**↑**
FSGS-8	**↑**	**↑**
FSGS-9	**↑**	**↑**
FSGS-10	**↑**	**↑**

*Abbreviation*: *=* staining comparable to unaffected control kidney, ***↑*** increased staining compared to unaffected control kidney, *FSGS* focal segmental glomerulosclerosis, *ID* identification, *n/a* not available, *NICD* Notch1 intracellular domain, *SIOD* Schimke immuno-osseous dysplasia, *Tx* transplant

^a^No more tissue sections were available for analysis

### Markers of Wnt and Notch pathway activation are not increased a 15-week-gestation SMARCAL1-deficient kidney

To determine whether pathologically increased Wnt and Notch pathway signaling in SIOD begins prenatally, we performed indirect immunofluorescence for unphosphorylated β-catenin and NICD in a 15-week-gestation SMARCAL1-deficient kidney and age-matched unaffected kidneys. The SMARCAL1-deficient fetal kidney expressed comparable levels of unphosphorylated β-catenin and NICD to the age-matched controls in both S-shaped bodies and developing glomeruli (Additional file 1: Figure S6 and Figure S7). In agreement with these findings, expression analysis of several Wnt and Notch target genes in the SMARCAL1-deficient fetal kidney and age-matched controls demonstrated comparable expression levels (Additional file 1: Figure S8).

### Markers of Wnt and Notch pathway activation are not increased in the transplanted kidney of an SIOD patient

Our previous studies have shown that the renal disease of SIOD is cell autonomous [5, 32]; therefore, we hypothesized that if the increased glomerular levels of unphosphorylated β-catenin and NICD are potentially causative of the renal disease in SIOD, then the levels of unphosphorylated β-catenin and NICD are not increased in renal grafts of SIOD patients. To test this hypothesis, we performed indirect immunofluorescence for unphosphorylated β-catenin and NICD in the transplanted kidney of an SIOD patient and observed a staining pattern and intensity similar to that of unaffected controls for unphosphorylated β-catenin and NICD (Additional file 1: Figure S3, Figure S4, and Figure S5).

### *Drosophila Marcal1* genetically interacts with the Wnt and Notch signaling pathways

To assess whether the upregulation of the Wnt and Notch signaling pathways is a genetic consequence of SMARCAL1 deficiency and not simply an end product of the tissue pathology, we performed overexpression and loss-of-function genetic screens in *Drosophila*. By assessing the suppression or enhancement of ectopic wing veins induced by *Marcal1* overexpression [13], we found that both Wnt and Notch pathway genes genetically interacted with *Marcal1* (Additional file 1: Table S7, Table S8, Figure S9, Figure S10, and Figure S11).

**Fig. 4:**
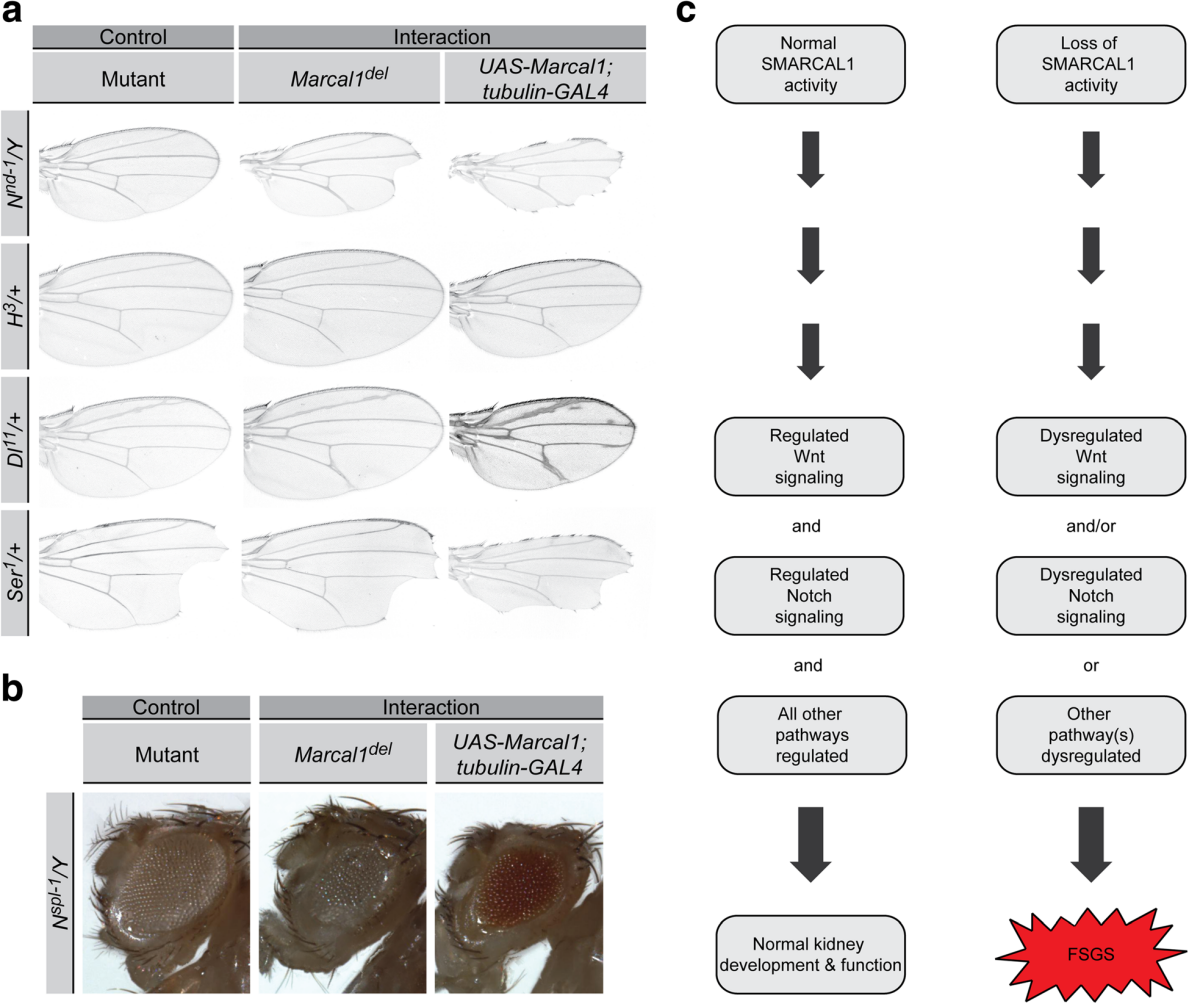
Genetic interaction of *Marcal1* loss and gain with Notch pathway mutant alleles and model. **a** Representative wings of the mutant allele of interest (*left column*), the mutant allele in the *Marcal1* loss-of-function background (*middle column*), and the mutant allele in the *Marcal1* overexpression background (*right column*). Hairless (*H*), Delta (*Dl*), and Serrate (*Ser*) are dominant alleles on chromosome 3. Although both heterozygous males and females were assessed, representative wings from females are shown. The *N* ^*nd-1*^ allele is a homozygous viable allele of Notch on chromosome 1. Although both homozygous females and hemizygous males were assessed, representative wings from hemizygous males are shown. **b** Representative eyes of the mutant allele *N* ^*spl-1*^ (*left*), the mutant allele in the *Marcal1* loss-of-function background (*middle*), and the mutant allele in the *Marcal1* overexpression background (*right*). The *N* ^*spl-1*^ allele is a homozygous viable allele of Notch on chromosome 1. Although both homozygous females and hemizygous males were assessed, representative eyes from hemizygous males are shown. **c** Model of renal disease pathogenesis in SIOD. Normal SMARCAL1 activity leads to regulated signaling of pathways and normal kidney development and function, whereas loss of SMARCAL1 activity leads to dysregulated Wnt and/or Notch signaling and in turn causes FSGS

To confirm these interactions, we performed the reciprocal analysis, i.e., analysis of the suppression or enhancement of phenotypes associated with Wnt and Notch pathway mutants. For the well-characterized wing, eye, and bristle phenotypes of Notch pathway mutants, *Marcal1* loss and gain suppressed or enhanced phenotypes for Notch (*N*) mutants, Delta (*Dl*) mutants, Hairless (*H*) mutants, and a fringe (*fng*) mutant (Fig. 4a and b, Additional file 1: Table S9 and Figure S12). No genetic interaction was observed between *Marcal1* loss or gain and a Serrate (*Ser*) mutant (Fig. 4a and Additional file 1: Table S9).

## Discussion

Herein we identify increased signaling of the Wnt and Notch pathways as potential causes for the renal disease in SIOD. Most SIOD kidneys exhibited increased levels of unphosphorylated β-catenin and NICD respectively indicating increased Wnt and Notch pathway activity. Similarly, most isolated FSGS kidneys had upregulated unphosphorylated β-catenin and NICD. The failure to observe increased unphosphorylated β-catenin and NICD in the renal graft of an SIOD patient suggests that these molecular findings are inherent to the diseased kidney and not induced from outside of the kidney. The genetic interaction between *Marcal1* and the Wnt and Notch pathway genes in *Drosophila* suggests that that the altered signaling of these pathways is a direct or indirect consequence of SMARCAL1 deficiency.

The consistency of increased markers for both activation of the Wnt and Notch pathways in both SIOD and isolated FSGS control kidneys suggests that activation of both pathways underlie the renal disease of SIOD and isolated FSGS (Fig. 4c). Activation of both pathways is not essential for induction of SIOD renal disease or isolated FSGS, however, because a few samples showed activation of only one or neither of these pathways (Fig. 4c).

Based on our observations in the 15-week-gestation fetal kidney, the potentially pathological activation of Wnt and Notch signaling in the SIOD kidneys appears to arise after this stage of renal development. Further studies are required to define precisely the timing of the pathological activation of these pathways.

Although the Notch pathway gene expression changes were not identified in the KEGG pathway analysis of the transcriptome, the high level of crosstalk between the Wnt and Notch signaling pathways [33], and their role in kidney development and disease prompted us to also investigate the upregulation of the Notch pathway as a potential cause for the FSGS in SIOD. Possible reasons for the transcriptome analysis not detecting the upregulation of the Notch pathway include pathway size bias inherent to KEGG pathway analysis (the Wnt signaling pathway includes 141 genes, whereas the Notch signaling pathway includes 48 genes) and tissue heterogeneity.

The mechanism by which SMARCAL1 deficiency gives rise to tissue-specific changes in gene expression is incompletely understood. It could arise from a direct consequence of SMARCAL1 deficiency on the DNA structure of a gene or of the genes encoding the transcriptional regulators of that gene. Consistent with this, we previously observed that SMARCAL1 homologues bind transcriptionally active chromatin and modulate gene expression [13]. Sharma et al. (2015) recently showed that the bovine orthologue of SMARCAL1 negatively and directly regulates the transcription of *MYC* by altering the conformation of its promoter [34]. Alternatively, because stalled replication forks induce epigenetic changes that alter gene expression [35, 36], impedance of DNA replication fork restart by SMARCAL1 deficiency might contribute to the changes in gene expression. Consistent with the latter possibility, we recently observed hypermethylation of the *IL7R* promoter in the T cells of SIOD patients [19]; reduced *IL7R* expression in human CD8^+^ T cells is associated with hypermethylation of the *IL7R* promoter [37].

A limitation of the study was the use of whole kidney to profile differential gene expression in an SIOD kidney. Given that the primary lesion is limited to the glomeruli, the affected tissue represents a small fraction of the total tissue. Although several human gene expression studies on FSGS have used isolated glomeruli [38, 39], others have successfully used renal biopsies [40]. Similar to other human gene expression studies of FSGS [38–40], the expression of podocyte-specific genes including *NPHS1*, *NPHS2*, and *WT1* were downregulated in the SIOD kidney, and most of the KEGG pathways that were enriched in our list of upregulated genes were also enriched in the prior studies, including the Wnt signaling pathway [38].

A second limitation of the study was that only unphosphorylated β-catenin and nuclear NICD were examined by immunofluorescence as measures of pathway activation. This constraint arose secondary to limited tissue. We selected these proteins because they are the primary effectors of and activation markers for the canonical Wnt and Notch signaling pathways. However, Wnt signaling has canonical and non-canonical pathways, and there is also Wnt-independent β-catenin activation [41]. Notch signaling also has canonical and non-canonical pathways as well as three Notch receptors in addition to Notch1 [42]. Our findings nonetheless set a precedent for future studies examining the pathogenesis of renal disease in SIOD.

## Conclusions

In summary, our findings show that the Wnt and Notch pathways are upregulated in the SIOD patient kidney and that *Marcal1*, the *Drosophila SMARCAL1* homologue, genetically interacts with Wnt and Notch pathway genes. Based on these findings, the renal disease of SIOD is yet another clinically distinctive feature of SIOD likely arising through alterations of gene expression.

## Additional file

Additional file 1: Supplementary Methods, Tables, and Figures. (PDF 45904 kb)

## Abbreviations

ACV: Anterior crossvein
DAPI: 4', 6-diamidino-2-phenylindole
DAVID: Database for annotation, visualization, and integrated discovery
FFPE: Formalin-fixed paraffin-embedded
FPKM: Fragments per kilobase per million mapped reads
FSGS: Focal segmental glomerulosclerosis
KEGG: Kyoto Encyclopedia of Genes and Genomes
NICD: Notch1 intracellular domain
PBS: Phosphate-buffered saline
PCV: Posterior crossvein
qRT-PCR: Quantitative reverse transcription polymerase chain reaction
SIOD: Schimke immuno-osseous dysplasia
SMARCAL1: SWI/SNF-related, matrix-associated, actin-dependent regulator of chromatin, subfamily A-like 1
