# Supplementary material for: Increased Wnt and Notch signaling: a clue to the renal disease in Schimke immuno-osseous dysplasia?
Source: Orphanet J Rare Dis. 2016 Nov 5;11:149. doi: 10.1186/s13023-016-0519-7 (PMC5097426; doi:10.1186/s13023-016-0519-7)
Supplement: Additional file 1: — Supplementary Methods, Tables, and Figures. (PDF 45904 kb) [file 13023_2016_519_MOESM1_ESM.pdf]

Orphanet Journal of Rare Diseases

SUPPLEMENTARY INFORMATION

Increased Wnt and Notch signaling: A clue to the renal disease in Schimke immuno-osseous dysplasia?

Marie Morimoto, Clara Myung, Kimberly Beirnes, Kunho Choi, Yumi Asakura, Arend Bokenkamp, Dominique Bonneau, Milena Brugnara, Joel Charrow, Estelle Colin, Amira Davis, Georges Deschenes, Mattia Gentile, Mario Giordano, Andrew K. Gormley, Rajeshree Govender, Mark Joseph, Kory Keller, Evelyne Lerut, Elena Levtchenko, Laura Massella, Christy Mayfield, Behzad Najafian, David Parham, Jurgen Spranger, Peter Stenzel, Uluc Yis, Zhongxin Yu, Jonathan Zonana, Glenda Hendson, Cornelius F. Boerkoel

TABLE OF CONTENTS

METHODS…………3

TABLE S1…………8

TABLE S2…………9

TABLE S3…………11

TABLE S4…………12

TABLE S5…………15

TABLE S6…………16

TABLE S7…………17

TABLE S8…………18

TABLE S9…………19

FIGURE S1…………23

FIGURE S2…………24

FIGURE S3…………25

FIGURE S4…………26

FIGURE S5…………27

FIGURE S6…………28

FIGURE S7…………29

FIGURE S8…………30

FIGURE S9…………31

FIGURE S10…………33

FIGURE S11…………34

FIGURE S12…………35

REFERENCES…………36

SUPPLEMENTARY METHODS

Cell culture

Unaffected primary human dermal fibroblasts (GM00942, Coriell Institute for Medical Research, Camden, NJ, USA) and HeLa cells (American Type Culture Collection, Manassas, VA, USA) were cultured in high glucose Dulbecco's Modified Eagle Medium (DMEM, Gibco, Thermo Fisher Scientific, Waltham, MA, USA) supplemented with 10% fetal bovine serum (Gibco, Thermo Fisher Scientific, Waltham, MA, USA) and 1× antibiotic-antimycotic (Gibco, Thermo Fisher Scientific, Waltham, MA, USA) at 37°C and 5% CO_2. Cells were grown to approximately 90% confluence.

Wnt3a treatment

Prior to the beginning of the time course, the medium was replaced with low serum medium containing 0.5% fetal bovine serum for 48 hours. The dermal fibroblasts and HeLa cells were treated with 100 ng/ml Wnt3a (R&D Systems, Minneapolis, MN, USA) for 4 or 6 hours, respectively. Untreated cells were treated with the same volume of the vehicle solution. The cells were then pelleted, formalin-fixed, and paraffin-embedded using standard procedures for use as controls for the β-catenin immunofluorescence staining.

Quantification of β-catenin immunofluorescence

ImageJ (U.S. National Institutes of Health, Bethesda, MD, USA) was used to quantify and compare the glomerular β-catenin signal between the various kidney tissue sections. Representative images were captured using a 20× Plan-APOCHROMAT objective and a 10×/23 mm eyepiece (Carl Zeiss, Toronto, ON, Canada). Though this setup provides a 1,150 μm circular

optical field of view, the AxioCam MR microscope camera captures images of approximately 448 μm × 336 μm. The analyzed images are presented as the overview images in Figure 2 and Additional file 1: Figure S3. Using ImageJ, the glomeruli were outlined and the area and integrated density were measured, as well as the mean fluorescence of three adjacent background regions. The corrected total fluorescence (CTF = integrated density - (glomerular area × mean fluorescence of background readings) was calculated for each glomerulus. Where there was more than one glomerulus present in the image, an average CTF of all glomeruli was calculated. Data were plotted as box and whisker plots and analyzed by the 2-tailed Student's *t*-test. A *p* value of less than 0.05 was considered statistically significant. The Bonferroni correction was applied to correct for multiple comparisons.

Drosophila melanogaster genetic screen to determine the effect of Wnt and Notch mutant alleles on the _Marcal1_ overexpression phenotype

Drosophila wings have five longitudinal veins (L1, L2, L3, L4, and L5) plus anterior and posterior cross veins (ACV and PCV) (Additional file 1: Figure S1B). The overexpression of *Marcal1* induces an ectopic vein parallel and anterior to L2, an ectopic vein extending laterally from the PCV, a partially missing or completely absent ACV or PCV, and distal bending or splitting of longitudinal veins L2, L4, and L5 (Additional file 1: Figure S1C) [1]. These wing vein alterations are dependent on enzymatic activity since overexpression of the enzymatically inactive mutant $Marcal1^{K275R}$ does not alter wing venation [1]. We screened for Wnt and Notch alleles that lead to the suppression or enhancement of the ectopic wing veins induced by the overexpression of *Marcal1* [1].

Crosses for analyzing the genetic interaction between the *Marcal1* gene with genes encoding for components of the Wnt and Notch signaling pathways were maintained at 25°C for three days. The crosses were then transferred to 28°C. Upon eclosion, the desired F_1 progeny were selected and their wings were mounted, imaged, and assessed as previously described [1].

Ectopic wing veins observed in the F_1 progeny of the crosses were scored according to the following guidelines. For each of the features scored, a phenotype resembling the wild type wing was given a score of 0. Ectopic veins parallel and anterior to the L2 longitudinal vein were given a score of 0 to 2 based on the length of the ectopic vein. The distal portion of the L2 vein was given a score of 0 or 1 based on the absence or presence of bending or splitting. The distal portions of the L4 and L5 veins were given a score of 0 to 4 proportionate to the degree of deviation from the wild type phenotype. Ectopic veins extending perpendicularly from the PCV were given a score of 0 to 3 based on the length of the ectopic vein. A partially or completely absent ACV or PCV were each given a score of 1. Representative images for these phenotypes and their respective scores are present in Additional file 1: Figure S1C. The reference wing vein phenotype was determined by crossing *Marcal1* overexpression flies to w^{1118} mutants of three genetic backgrounds; the scores from these crosses were averaged to provide a reference score. To determine whether there was any non-specific interaction between the various mutant alleles and the GAL4-UAS system, all mutant lines were crossed to the *C96-GAL4, UAS-Hrs/MKRS* transgenic line and the degree of wing margin scalloping in the desired F_1 progeny was scored [2]. Any mutant alleles that interacted with the GAL4-UAS system (i.e., those that enhanced or suppressed the wing margin scalloping phenotype of the *C96-GAL4, UAS-Hrs/MKRS* transgenic line) were excluded; all mutant alleles presented here had no detectable non-specific interactions with the GAL4-UAS system.

Ten or more wings were analyzed for each cross and scored by two independent readers. Scores for each cross were compared to the reference scores to determine whether the wing vein phenotype was suppressed or enhanced. Where there was a discrepancy between the first two reads, a third read was completed by C. F. B.

Drosophila melanogaster genetic screen to determine the effect of _Marcal1_ loss and gain on Notch mutant phenotypes

Several Notch mutant alleles cause phenotypes that manifest in the wing, eye, and bristle. We screened for the suppression or enhancement of these phenotypes in the context of *Marcal1* loss or gain. For the loss-of-function screen, selected Notch pathway mutant alleles from the Bloomington *Drosophila* Stock Center (Bloomington, IN, USA) were crossed into the *Marcal1* loss-of-function background. These crosses were carried out and maintained at 20°C. For the overexpression screen, selected Notch pathway mutant alleles were crossed into the *Marcal1* overexpression background as detailed above. Upon eclosion, the desired F_1 progeny were selected, and the relevant phenotype analyzed. Since several of the alleles are temperature-dependent, the Notch mutant phenotypes were also assessed at 28°C.

A minimum of 40 wings was scored for each genotype where the suppression or enhancement of a wing phenotype was being assessed. Notch alleles N^{nd-1} and N^{nd-3} exhibit wing notching in homozygous females and hemizygous males; all Delta alleles in this study exhibit deltas, wing vein thickening, and ectopic veins in heterozygous flies; the Serrate allele Ser^{1} exhibits serrated wings in heterozygous flies; the fringe allele fng^{13} occasionally exhibits wing notching in heterozygous flies; and all Hairless alleles in this study exhibit shortened

longitudinal veins in heterozygous flies as previously described [3-5]. The presence or absence of the phenotype of interest was scored for each wing.

Eighty bristles of the relevant type were scored for each genotype where the suppression or enhancement of a bristle phenotype was being assessed. Notch allele N^{spl-1} exhibits missing, double, or ectopic anterior and posterior scutellar bristles in homozygous females and hemizygous males, while all Hairless alleles in this study exhibit missing bristles on the head and notum in heterozygous flies as previously described [5, 6]. A total of 80 anterior and posterior scutellar bristles were scored for the N^{spl-1} allele, while 80 bristles of each type on the notum were scored for the Hairless alleles. The presence or absence of the phenotype of interest was scored for each bristle type.

Eighty eyes were scored for each genotype where the suppression or enhancement of an eye phenotype was being assessed. The Notch allele N^{spl-1} exhibits rough and reduced eyes in homozygous females and hemizygous males as previously described [7].

Wings exhibiting a blistered phenotype and eyes exhibiting a rough and reduced eye phenotype were imaged using an MZ16 Stereomicroscope (Leica Microsystems Inc., Concord, ON, Canada).

SUPPLEMENTARY TABLES

Table S1. *SMARCAL1* mutations identified in SIOD patients included in this study.

Patient ID	Sex	*SMARCAL1* mutations	
		**Nucleotide change**	**Predicted amino acid change**
SD4b	M	c.[410delA];[1930C>T]	p.[(Q137Rfs*3)];[(R644W)]
SD26	M	c.[1190delT];[2542G>T]	p.[(L397Rfs*40)];[(E848X)]
SD51	F	c.[2459G>A];[2542G>T]	p.[(R820H)];[(E848X)]
SD60	M	c.[2542G>T];[2542G>T]	p.[(E848X)];[(E848X)]
SD79	F	c.[2459G>A];[?][1]	p.[(R820H)];[(?)]
SD120	M	c.[2291G>A];[2542G>T]	p.[(R764Q)];[(E848X)]
SD121	F	c.[1382G>A];[2542G>T]	p.[(G461D)];[(E848X)]
SD131	M	c.[1026C>A];[2264T>G]	p.[(Y342X)];[(I755S)]
SD133b	F	c.[863-2A>G];<br>[2343_2347delGCTGT]	p.[(M288_D366delinsN)];<br>[(L782Hfs*14)]
SD146	F	c.[1642_1644delATT];<br>[1642_1644delATT]	p.[(I548del)];[(I548del)]

[1][?] represents an allele with a noncoding *SMARCAL1* mutation as previously described by Clewing *et al*.

Abbreviations: F, female; ID, identification; M, male; SIOD, Schimke immuno-osseous dysplasia; *SMARCAL1*, SWI/SNF-related matrix-associated actin-dependent regulator of chromatin, subfamily A-like 1.

Table S2. Human tissue samples used in the study.

Sex	Age at sampling	Tissue	Sample type	Studies	Comments
M	3 years	Kidney	RNA from frozen tissue	Fig. 1	Unaffected control
M	5.4 years	Kidney	RNA from frozen tissue	Fig. 1	SIOD patient SD120
F	7 years	Kidney	FFPE	Figs. 2, 3, S. Fig. 2	Unaffected control
M	8 years	Kidney	FFPE	Figs. 2, 3, S. Fig. 2	SIOD patient SD4b
M	4 years	Kidney	FFPE	Figs. 2, 3, S. Fig. 2	SIOD patient SD26
F	< 10 years	Kidney	RNA from cultured cells	n/a (microarray)	FSGS patient
F	4.8 years	Kidney	RNA from cultured cells	n/a (microarray)	SIOD patient SD51
M	13.7 years	Kidney	FFPE	Figs. 2, 3, S. Fig. 2	SIOD patient SD60
F	10 years	Kidney	FFPE	Figs. 2, 3, S. Fig. 2	SIOD patient SD79
M	5.4 years	Kidney	FFPE	Figs. 2, 3, S. Fig. 2	SIOD patient SD120
F	3 years	Kidney	FFPE	Figs. 2, 3, S. Fig. 2	SIOD patient SD121
M	3.8 years	Kidney	FFPE	Fig. 3	SIOD patient SD131
F	2 years	Kidney	FFPE	Figs. 2, 3, S. Fig. 2	SIOD patient SD146
F	16 years	Skin	FFPE	Fig. 3	Positive control for Notch1 IF
F	17 years	Adenoma	FFPE	S. Fig. 1	Positive control for β-catenin IF
F	3 years	Kidney	FFPE	S. Figs. 2-4	Unaffected control
F	14 years	Kidney	FFPE	S. Figs. 2-4	Unaffected control
M	13.7 years	Tx Kidney	FFPE	S. Figs. 2-4	SIOD patient SD60
?	?	Kidney	FFPE	S. Figs. 2-4	FSGS patient FSGS-1
?	?	Kidney	FFPE	S. Figs. 2-4	FSGS patient FSGS-2
?	?	Kidney	FFPE	S. Figs. 2-4	FSGS patient FSGS-3
?	?	Kidney	FFPE	S. Figs. 2-4	FSGS patient FSGS-4
?	?	Kidney	FFPE	S. Figs. 2-4	FSGS patient FSGS-5
?	?	Kidney	FFPE	S. Figs. 2-4	FSGS patient FSGS-6
?	?	Kidney	FFPE	n/a	FSGS patient FSGS-7, no glomeruli in this biopsy
?	?	Kidney	FFPE	S. Figs. 2-4	FSGS patient FSGS-8
?	?	Kidney	FFPE	S. Figs. 2-4	FSGS patient FSGS-9
?	?	Kidney	FFPE	S. Figs. 2-4	FSGS patient FSGS-10

Sex	Age at sampling	Tissue	Sample type	Studies	Comments
M	15 weeks gestation	Kidney	FFPE	S. Figs. 5, 6	Unaffected control H-25194
F	15 weeks gestation	Kidney	FFPE	S. Figs. 5, 6	Unaffected control H-25217
F	15 weeks gestation	Kidney	FFPE	S. Figs. 5, 6	SMARCAL1-deficient fetus SD133b
M	15 weeks gestation	Kidney	RNA from FFPE tissue	S. Fig. 7	Unaffected control H-25194
M	15 weeks gestation	Kidney	RNA from FFPE tissue	S. Fig. 7	Unaffected control AE14 3118
M	15 weeks gestation	Kidney	RNA from FFPE tissue	S. Fig. 7	Unaffected control AE14 3138
F	15 weeks gestation	Kidney	RNA from FFPE tissue	S. Fig. 7	Unaffected control AE14 3158
M	15 weeks gestation	Kidney	RNA from FFPE tissue	S. Fig. 7	Unaffected control AE15 3040
F	15 weeks gestation	Kidney	RNA from FFPE tissue	S. Fig. 7	SMARCAL1-deficient fetus SD133b

Abbreviations: ?, unknown; F, female; Fig., Figure; Figs., Figures; FFPE, formalin-fixed paraffin-embedded; FSGS, focal segmental glomerulosclerosis; IF, immunofluorescence; M, male; n/a, not applicable; RNA, ribonucleic acid; SIOD, Schimke immuno-osseous dysplasia; S.; Supplementary; Tx, transpl

Table S3. Primer sequences used in this study.

Primer	Sequence
***Drosophila* Marcal1 overexpression**	
Gapdh2-F	5′-ATCGTCGAGGGTCTGATGAC-3′
Gapdh2-R	5′-TCAGCTTCACGAACTTGTCG-3′
Marcal1-2F	5′-AAGTGCTACGATGGCCAAAC-3′
Marcal1-2R	5′-GCCTGATCCGTGAGTCTTTT-3′
**Human Wnt target gene expression**	
GAPDH-F	5′-CTTTTGCGTCGCCAGCCGAG-3′
GAPDH-R	5′-GGTGACCAGGCGCCCAATACG-3′
AXIN2-F	5′-TAACCCCTCAGAGCGATGGA-3′
AXIN2-R	5′-AACCTCCTCTCTTTTACAGCAGG-3′
CCND1-F	5′-CTTCAAATGTGTGCAGAAGGAGG-3′
CCND1-R	5′-CTCGCAGACCTCCAGCATC-3′
CCND2-F	5′-AGCAGGATGAGGAAGTGAGC-3′
CCND2-R	5′-GACAATCCACGTCTGTGTTGG-3′
JUN-F	5′-AGGGTCCGCACTGATCCGCT-3′
JUN-R	5′-CTCGGAGTCCGCAGGCGAAC-3′
**Human Notch target gene expression**	
GAPDH-F	5′-CTTTTGCGTCGCCAGCCGAG-3′
GAPDH-R	5′-GGTGACCAGGCGCCCAATACG-3′
HES1-F	5′-AGAATAAATGAAAGTCTGAGCCAGC-3′
HES1-R	5′-ATGCCGCGAGCTATCTTTCT-3′
HES2-F	5′-GACCTCGGTTTCCCTTTGCG-3′
HES2-R	5′-CTTCAGGCTCTTGCGCAGC-3′
HEY1-F	5′-GGATCTGCTAAGCTAGAAAAAGCC-3′
HEY1-R	5′-AAGTAACCTTTCCCTCCTGCC-3′
HEY2-F	5′-AGAACAATTACTCGGGGCAAAGT-3′
HEY2-R	5′-TCCCTCTCCTTTTCTTTCTTGCC-3′

Abbreviations: *AXIN2*, axin 2; *CCND1*, cyclin D1; *CCND2*, cyclin D2; F, forward; *GAPDH*, glyceraldehyde 3-phosphate dehydrogenase; *HES1*, hes family bHLH transcription factor 1; *HES2*, hes family bHLH transcription factor 2; *HEY1*, hes related family bHLH transcription factor with YRPW motif 1; *HEY2*, hes related family bHLH transcription factor with YRPW motif 2; *JUN*, Jun proto-oncogene; R, reverse.

Table S4. Significantly enriched KEGG pathway terms of upregulated genes (log_2 fold change > 1) in an SIOD kidney compared to a sex-matched unaffected control kidney.

Term	Genes[1]	%[2]	Fold enrichment[1]	*p* value[4]
hsa04512: ECM-receptor interaction	*AGRN*, *CD36*, *CD44*, *CD47*, *COL1A1*, *COL3A1*, *COL4A1*, *COL4A2*, *COL5A1*, *COL5A2*, *COL6A1*, *COL6A2*, *COL6A3*, *COMP*, *FN1*, *HSPG2*, *ITGA1*, *ITGA2*, *ITGA4*, *ITGA5*, *ITGA11*, *ITGB1*, *ITGB4*, *ITGB6*, *LAMA2*, *LAMB1*, *LAMBC1*, *LAMC2*, *LAMC3*, *SDC3*, *THBS1*, *THBS2*, *TNC*, *VWF*	1.5	2.9	1.5E-06
hsa04510: Focal adhesion	*ACTN1*, *AKT2*, *AKT3*, *BCL2*, *CCND2*, *COL1A1*, *COL3A1*, *COL4A1*, *COL4A2*, *COL5A1*, *COL5A2*, *COL6A1*, *COL6A2*, *COL6A3*, *COMP*, *FLNA*, *FLNB*, *FN1*, *HGF*, *IGF1*, *IGF1R*, *ITGA1*, *ITGA2*, *ITGA4*, *ITGA5*, *ITGA11*, *ITGB1*, *ITGB4*, *ITGB6*, *JUN*, *LAMA2*, *LAMB1*, *LAMC1*, *LAMC2*, *LAMC3*, *MAPK10*, *MYL9*, *MYLK*, *PDGFA*, *PDGFC*, *PDGFRA*, *PDGFRB*, *PIK3CD*, *PIK3R1*, *PPP1R12A*, *PTEN*, *PXN*, *RAC1*, *RAC2*, *RAPGEF1*, *SHC1*, *SRC, THBS1*, *THBS2*, *TNC*, *VAV1*, *VEGFC*, *VWF*	2.6	2.0	1.1E-05
hsa05322: Systemic lupus erythematosus	*ACTN1*, *C1QA*, *C1QB*, *C1QC*, *C1S*, *C1R*, *C3*, *C7*, *CD40*, *CD86*, *CTSG*, *FCGR1A*, *FCGR2A*, *FCGR2C*, *FCGR3A*, *FCGR2B*, *H2AFX*, *H2AFY*, *HIST1H2AB*, *HIST1H2AE*, *HIST1H2AG*, *HIST1H2AH*, *HIST1H2AI*, *HIST1H2AJ*, *HIST1H2AL*, *HIST1H2AM*, *HIST1H2BB*, *HIST1H2BE*, *HIST1H2BH*, *HIST1H2BJ*, *HIST1H2BL*, *HIST1H2BM*, *HIST1H2BN*, *HIST1H2BO*, *HIST1H3B*, *HIST1H3C*, *HIST1H3D*, *HIST1H3F*, *HIST1H3G*, *HIST1H3H*, *HIST1H3I*, *HIST1H3J*, *HIST1H4A*, *HIST1H4B*, *HIST1H4D*, *HIST1H4H*, *HIST1H4I*, *HIST1H4J*, *HIST1H4K*, *HIST1H4L*, *HIST2H2AB*, *HIST2H2AC*, *HIST2H3A*, *HIST2H3D*, *HIST2H4B*, *HIST3H2A*, *HIST3H2BB*, *HLA-DQA1*, *HLA-DRB5*[5]	1.6	2.6	1.3E-05
hsa05200: Pathways in cancer	*ABL1*, *AKT2*, *AKT3*, *APC*, *AXIN1*, *BCL2*, *BMP4*, *CASP3*, *CDKN1B*, *CDK6*, *CEBPA*, *CKS1B*, *COL4A1*, *COL4A2*, *CREBBP*, *CSF1R*, *CTBP1*, *FADD*, *FGFR3*, *FGF7*, *FN1*, *FOS*, *FZD2*, *FZD6*, *FZD7*, *FZD8*, *HGF*, *HIF1A*, *HSP90AA1*, *HSP90AB1*, *IGF1*, *IGF1R*, *ITGA2*, *ITGB1*, *JAK1*, *JUN*, *LAMA2*, *LAMB1*, *LAMC1*, *LAMC2*, *LAMC3*, *LEF1*, *MAPK10*, *MECOM*, *MITF*, *MMP2*, *MYC*, *NCOA4*, *PDGFA*, *PDGFRA*, *PDGFRB*, *PIK3CD*, *PIK3R1*, *PLCG2*, *PML*, *PPARD*, *PPARG*, *PTCH1*, *PTEN*, *RAC1*, *RAC2*, *RALBP1*, *RALGDS*, *RARA*,	3.6	1.7	3.6E-05

Term	Genes[1]	%[2]	Fold enrichment[1]	*p* value[4]
	RASSF5, *RUNX1*, *SMAD4*, *SPI1*, *STAT1*, *STAT3*, *STK4*, *TCF7L2*, *TFG*, *TGFB1*, *TGFB2*, *TGFBR2*, *VEGFC*, *WNT4*, *WNT5A*, *WNT9B*, *WNT10A*			
hsa04670: Leukocyte transendothelial migration	*ACTN1*, *CD99*, *CLDN3*, *CLDN5*, *CLDN7*, *CLDN10*, *CLDN11*, *CLDN14*, *CLDN15*, *CLDN16*, *CLDN19*, *CTNND1*, *CXCL12*, *CXCR4*, *CYBA*, *CYBB*, *EZR*, *GNAI2*, *ITGA4*, *ITGAM*, *ITGB1*, *ITGB2*, *JAM2*, *JAM3*, *MMP2*, *MYL9*, *NCF2*, *NCF4*, *PIK3CD*, *PIK3R1*, *PLCG2*, *PXN*, *RAC1*, *RAC2*, *RASSF5*, *VAV1*, *VCAM1*	1.7	2.2	5.2E-04
hsa04310: Wnt signaling pathway	*APC*, *AXIN1*, *CCND2*, *CREBBP*, *CSNK1E*, *CTBP1*, *DAAM1*, *FZD2*, *FZD6*, *FZD7*, *FZD8*, *JUN*, *LEF1*, *MAP3K7*, *MAPK10*, *MMP7*, *MYC*, *NFAT5*, *NFATC3*, *NFATC4*, *NKD2*, *PPARD*, *PPP2R5A*, *PPP2R5C*, *PPP2R5D*, *PPP3CA*, *PRICKLE1*, *PRKACA*, *PRKACB*, *PSEN1*, *RAC1*, *RAC2*, *SFRP1*, *SFRP2*, *SMAD4*, *TBL1X*, *TBL1XR1*, *TCF7L2*, *VANGL2*, *WNT4*, *WNT5A*, *WNT9B*, *WNT10A*	1.9	2.0	1.1E-03
hsa05210: Colorectal cancer	*AKT2*, *AKT3*, *APC*, *AXIN1*, *BCL2*, *CASP3*, *FOS*, *FZD2*, *FZD6*, *FZD7*, *FZD8*, *JUN*, *IGF1R*, *LEF1*, *MAPK10*, *MYC*, *PDGFRA*, *PDGFRB*, *PIK3CD*, *PIK3R1*, *RAC1*, *RAC2*, *RALGDS*, *SMAD4*, *TCF7L2*, *TGFB1*, *TGFB2*, *TGFBR2*	1.2	2.4	3.2E-03
hsa04666: Fc gamma R-mediated phagocytosis	*AKT2*, *AKT3*, *ASAP1*, *DNM1*, *DNM1L*, *DNM2*, *DOCK2*, *FCGR1A*, *FCGR2A*, *FCGR2B*, *FCGR2C*, *FCGR3A*, *GAB2*, *GSN*, *HCK*, *INPP5D*, *MARCKS*, *MARCKSL1*, *PIK3CD*, *PIK3R1*, *PIP4K2B*, *PIP5K1A*, *PLA2G4A*, *PLA2G4B*, *PLCG2*, *PLPP2*, *RAC1*, *RAC2*, *SYK*, *VAV1*, *WASF2*[6]	1.3	2.2	4.7E-03
hsa04514: Cell adhesion molecules	*CD4*, *CD40*, *CD58*, *CD86*, *CD99*, *CD276*, *CDH3*, *CLDN3*, *CLDN5*, *CLDN7*, *CLDN10*, *CLDN11*, *CLDN14*, *CLDN15*, *CLDN16*, *CLDN19*, *CNTNAP1*, *HLA-DRB5*, *HLA-DQA1*, *ICAM2*, *ICOSLG*, *ITGA4*, *ITGAM*, *ITGB1*, *ITGB2*, *JAM2*, *JAM3*, *NCAM1*, *NEGR1*, *NFASC*, *NRXN2*, *PVR*, *SDC3*, *VCAM1*, *VCAN*	1.6	1.9	4.4E-02

[1]The genes that are involved in the individual KEGG pathway term.
[2]The number of genes involved in a given term divided by the total number of input genes (i.e., 2,241 genes).
[3]The magnitude of enrichment of a given term represented by the ratio of the proportion of genes involved in the term within the input genes and of the proportion of genes involved in the term within the human genome.
[4]Modified Fisher's Exact *p* values, corrected for multiple comparisons by the Bonferroni method, representing the significance of enrichment of a given term.
[5]These 59 genes correspond to 60 RefSeq IDs and are represented by 36 unique DAVID gene IDs.
[6]These 31 genes correspond to 32 RefSeq IDs and are represented by 30 unique DAVID gene IDs.

Abbreviations: DAVID, Database for Annotation, Visualization, and Integrated Discovery; ECM, extracellular matrix; ID, identification; KEGG, Kyoto Encyclopedia of Genes and Genomes; SIOD, Schimke immuno-osseous dysplasia.

Table S5. Significant gene expression differences (fold change > 2) between an SIOD and a sex-matched unaffected control kidney measured by the WNT Signaling Pathway Plus PCR Array (PAHS-043Y).

Gene	Fold change	*p* value
**Upregulated genes**		
WNT7A	22.3	**
SFRP4	19.0	**
WNT10A	14.0	***
FRZB	12.3	**
WNT4	8.7	***
CCND2	7.9	***
WNT5A	7.6	***
VANGL2	6.9	**
MYC	6.5	***
DKK3	6.2	***
AXIN2	5.9	**
MMP7	5.6	***
WIF1	4.9	*
SFRP1	3.9	***
PRICKLE1	3.9	*
FZD7	3.5	**
FZD2	3.3	**
NFATC1	2.9	**
WNT9A	2.8	**
WNT5B	2.7	***
WNT6	2.7	*
LEF1	2.5	*
WNT2B	2.4	***
DAAM1	2.4	**
JUN	2.3	*
DKK1	2.2	*
TCF7	2.2	***
**Downregulated genes**		
MTFP1	-4.0	***
PITX2	-3.3	**
FZD5	-2.4	***

Abbreviations: *, $p < 0.05$; **, $p < 0.01$; ***, $p < 0.001$; SIOD, Schimke immuno-osseous dysplasia.

Table S6. Significant gene expression differences (fold change > 2) between an SIOD and a sex-matched unaffected control kidney measured by the Notch Signaling Pathway Plus PCR Array (PAHS-059Y).

Gene	Fold change	*p* value
**Upregulated genes**		
RUNX1	6.4	***
FIGF	3.8	***
UBD	3.5	**
CD44	3.2	**
MMP7	3.1	**
SHH	2.7	*
DTX1	2.6	*
IL2RA	2.6	**
HEYL	2.2	**
**Downregulated genes**		
SLC6A12	-13.8	**
SERPINA3	-7.3	*
PSENEN	-3.1	*
TFF1	-2.9	***
CCND1	-2.1	*

Abbreviations: *, $p < 0.05$; **, $p < 0.01$; ***, $p < 0.001$; SIOD, Schimke immuno-osseous dysplasia.

Table S7. Effect of Wnt pathway mutants on the ectopic wing veins induced by the overexpression of *Drosophila Marcal1*.

***Drosophila* gene**	**Allele**	**Effect on *Drosophila Marcal1* overexpression phenotype**
wg	*Sp-1*	0-S1
wg	*spd-1*	E1
wg	*l-16*	S1
wg	*1*	S2
wg	*l-17*	0-E1
wg	*l-12*	S2
dsh	*6*	S2
dsh	*1*	S2
dsh	*3*	S2
fz	*1*	Lethal
fz	*EY13696*	S2
sgg	*1*	E2-E3
sgg	*EP1576*	Lethal
Axn	*EY10228*	Lethal
Apc2	*d40*	0-S1
Apc2	*N175K*	S1-S2
Apc2<br>*Apc*	*N175K*<br>*Q8*	S1-S2
pan	*13a*	S1
pan	*2*	0-S1
pan	*3*	S1-S2
arm	*4*	S2
arm	*1*	S2
arm	*2*	S2
arm	*8*	S2
arm	*G0192*	S2

Abbreviations: S2, strong suppression; S1-S2, moderate suppression; S1, weak suppression; 0-S1, very weak suppression; 0, no enhancement or suppression; 0-E1, very weak enhancement; E1, weak enhancement; E1-E2, moderate suppression; E2, strong suppression; E2-E3, very strong enhancement.

Table S8. Effect of Notch pathway mutants on the ectopic wing veins induced by the overexpression of *Drosophila Marcal1*.

***Drosophila* gene**	**Allele**	**Effect on *Drosophila Marcal1* overexpression phenotype**
Dl	*3*	n/a[1]
Dl	*9*	n/a[1]
Dl	*11*	n/a[1]
Dl	*12*	n/a[1]
Dl	*13*	n/a[1]
Dl	*X*	n/a[1]
Dl	*14*	n/a[1]
Dl	*B2*	n/a[1]
Ser	*1*	S2
fng	*13*	S2
fng	*52*	S2
fng	*M69*	S1
N	*nd-1*	S2
N	*nd-3*	S2
N	*spl-1*	S1
Psn	*9*	S2
Psn	*143*	S2
Su(H)	*1*	S2
Su(H)	*2*	S2
Su(H)	*IB115*	S2
mam	*2*	S2
mam	*8*	S2
nej	*Q7*	S2
H	*1*	S1
H	*2*	S2
H	*3*	S2
CtBP	*03463*	0
CtBP	*87De-10*	S2
gro	*1*	S2
gro	*C105*	0-S1
HDAC1	*04556*	S1-S2
HDAC1	*303*	S2
HDAC1	*328*	S2
HDAC1	*def24*	S2

[1]Since several features of the *Dl* mutant phenotype and the *Marcal1* overexpression phenotype overlap, it was not possible to assess these wings.

Abbreviations: S2, strong suppression; S1-S2, moderate suppression; S1, weak suppression; 0-S1, very weak suppression; 0, no enhancement or suppression.

Table S9. Summary of the effect of *Marcal1* loss and gain on Notch pathway mutant allele phenotypes.

Gene	Allele	Mutant phenotype	Control penetrance		*Marcal1* loss-of-function penetrance[1]		*Marcal1* overexpression penetrance[1]	
			Male	Female	Male	Female	Male	Female
N	*spl-1*	Missing a sc bristle	3%	1%	1%	0%	**18%**	NA[3]
		Missing p sc bristle	51%	55%	**34%**	**40%**	**21%**	
		Double a sc bristle	25%	23%	**6%**	**1%**	18%	
		Double p sc bristle	1%	0%	3%	0%	3%	
		Ectopic a sc bristle	3%	15%	0%	0%	1%	
		Ectopic p sc bristle	0%	0%	0%	0%	1%	
		Rough and reduced eyes[2]	100%	100%	100%	100%	100%	
N	*nd-1*	Notched wings	12%	15%	**100%**	**91%**	**100%**	NA[3]
N	*nd-3*	Notched wings	26%	29%	**72%**	**45%**	**100%**	NA[3]
Dl	*3*	Delta wing veins	98%	100%	**100%**	**100%**	Blistered wings: 11%[4]	Blistered wings: 30%[4]
		Thickened and/or ectopic L2	40%	75%	**63%**	**96%**		
		Thickened and/or ectopic L3	13%	17%	10%	**33%**		
		Thickened L4 posterior to ACV	27%	56%	**83%**	**100%**		
		Thickened and/or ectopic PCV	21%	81%	**68%**	**100%**		
		Ectopic vein posterior to L5	21%	19%	**33%**	**83%**		
Dl	*11*	Delta wing veins	64%	38%	**79%**	**100%**	Blistered wings: 2%[4]	Blistered wings: 14%[4]
		Thickened and/or ectopic L2	100%	100%	**86%**	100%		
		Thickened L4 posterior to ACV	75%	48%	77%	**95%**		
		Thickened and/or ectopic PCV	7%	48%	**28%**	**93%**		
Dl	*12*	Delta wing veins	98%	63%	100%	**100%**	Blistered wings: 8%[4]	Blistered wings: 8%[4]
		Thickened and/or ectopic L2	95%	93%	92%	96%		
		Thickened and/or ectopic L3	0%	0%	6%	**50%**		
		Thickened L4 posterior to ACV	39%	65%	**25%**	71%		
		Thickened and/or ectopic PCV	16%	48%	**73%**	**96%**		
		Ectopic vein posterior to L5	0%	7%	6%	**52%**		
		Ectopic crossvein anterior to ACV	0%	2%	2%	**29%**		
Dl	*13*	Delta wing veins	24%	45%	**75%**	**93%**	Blistered	Blistered

Gene	Allele	Mutant phenotype	Control penetrance		*Marcal1* loss-of-function penetrance[1]		*Marcal1* overexpression penetrance[1]	
			Male	Female	Male	Female	Male	Female
		Thickened and/or ectopic L2	81%	79%	**13%**	**37%**	wings: 0%[4]	wings: 2%[4]
		Thickened L4 posterior to ACV	2%	9%	0%	0%		
		Thickened and/or ectopic PCV	2%	11%	**13%**	**37%**		
		Ectopic vein posterior to L5	4%	38%	3%	**17%**		
Dl	*14*	Delta wing veins	0%	28%	**100%**	**98%**	Blistered wings: 0%[4]	Blistered wings: 5%[4]
		Thickened and/or ectopic L2	39%	69%	**56%**	**76%**		
		Thickened and/or ectopic L3	0%	6%	7%	**50%**		
		Thickened L4 posterior to ACV	0%	0%	**28%**	**36%**		
		Thickened and/or ectopic PCV	0%	11%	**70%**	**100%**		
		Ectopic vein posterior to L5	0%	22%	0%	14%		
		Ectopic crossvein anterior to ACV	0%	0%	0%	**43%**		
Dl	*B2*	Delta wing veins	84%	92%	**100%**	100%	Blistered wings: 0%[4]	Blistered wings: 7%[4]
		Thickened and/or ectopic L2	82%	81%	**65%**	86%		
		Thickened and/or ectopic L3	10%	29%	10%	**57%**		
		Thickened L4 posterior to ACV	0%	32%	**23%**	**55%**		
		Thickened and/or ectopic PCV	23%	86%	25%	**76%**		
		Ectopic vein posterior to L5	3%	44%	**38%**	**62%**		
Dl	*X*	Delta wing veins	93%	100%	100%	100%	Blistered wings: 26%[4]	Blistered wings: 27%[4]
		Thickened and/or ectopic L2	89%	96%	**71%**	98%		
		Thickened and/or ectopic L3	7%	13%	5%	**50%**		
		Thickened L4 posterior to ACV	18%	51%	**80%**	**98%**		
		Thickened and/or ectopic PCV	39%	82%	**85%**	**94%**		
		Ectopic vein posterior to L5	7%	11%	**32%**	**96%**		
Ser	*1*	Serrated wing margin	100%	100%	100%	100%	100%	100%
fng	*13*	Loss of tissue from distal tip of wing	16%	6%	**1%**	1%	**0%**	0%
H	*1*	Shortened L4	1%	0%	lethal	lethal	0%	0%
		Shortened L5	100%	100%			**18%**	**0%**
		Missing u h bristle	73%	71%			**86%**	**39%**
		Missing l h bristle	14%	25%			9%	**4%**

Gene	Allele	Mutant phenotype	Control penetrance		*Marcal1* loss-of-function penetrance[1]		*Marcal1* overexpression penetrance[1]	
			Male	Female	Male	Female	Male	Female
		Missing p s bristle	16%	29%			**75%**	31%
		Missing a np bristle	0%	0%			5%	0%
		Missing p np bristle	0%	0%			1%	0%
		Missing a sa bristle	3%	1%			**14%**	**19%**
		Missing p sa bristle	68%	70%			**30%**	**24%**
		Missing a pa bristle	0%	0%			3%	0%
		Missing p pa bristle	31%	23%			**88%**	**44%**
		Missing a dc bristle	11%	30%			20%	**20%**
		Missing p dc bristle	4%	5%			1%	0%
		Missing a sc bristle	11%	6%			16%	9%
		Missing p sc bristle	1%	0%			3%	0%
H	*2*	Shortened L2	0%	0%	**20%**	**21%**	0%	0%
		Shortened L5	91%	92%	99%	95%	**52%**	**26%**
		Missing u h bristle	89%	94%	83%	**75%**	**59%**	**41%**
		Missing l h bristle	6%	6%	0%	3%	0%	4%
		Missing p s bristle	38%	41%	**14%**	**15%**	**25%**	**16%**
		Missing a np bristle	0%	0%	0%	0%	0%	0%
		Missing p np bristle	0%	0%	0%	0%	3%	0%
		Missing a sa bristle	1%	0%	0%	0%	**20%**	**25%**
		Missing p sa bristle	44%	44%	**3%**	**3%**	**4%**	**20%**
		Missing a pa bristle	0%	1%	0%	0%	1%	0%
		Missing p pa bristle	45%	41%	**8%**	**6%**	**31%**	**20%**
		Missing a dc bristle	80%	89%	**10%**	**14%**	**29%**	**54%**
		Missing p dc bristle	5%	0%	0%	1%	4%	5%
		Missing a sc bristle	0%	5%	0%	0%	**13%**	6%
		Missing p sc bristle	0%	1%	0%	0%	0%	0%
H	*3*	Shortened L2	0%	0%	3%	0%	0%	0%
		Shortened L5	99%	98%	97%	**84%**	**50%**	**10%**
		Missing u h bristle	74%	74%	70%	**64%**	**41%**	**34%**

Gene	Allele	Mutant phenotype	Control penetrance		*Marcal1* loss-of-function penetrance[1]		*Marcal1* overexpression penetrance[1]	
			**Male**	**Female**	**Male**	**Female**	**Male**	**Female**
		Missing l h bristle	1%	3%	0%	0%	5%	0%
		Missing p s bristle	31%	30%	**19%**	**10%**	**95%**	**71%**
		Missing a np bristle	1%	0%	0%	0%	0%	0%
		Missing p np bristle	0%	0%	0%	0%	0%	0%
		Missing a sa bristle	8%	1%	0%	0%	**24%**	**26%**
		Missing p sa bristle	45%	40%	**3%**	**1%**	**3%**	**8%**
		Missing a pa bristle	0%	0%	0%	0%	0%	0%
		Missing p pa bristle	59%	39%	**11%**	**4%**	59%	**23%**
		Missing a dc bristle	28%	58%	**4%**	**6%**	19%	**29%**
		Missing p dc bristle	5%	1%	0%	0%	3%	0%
		Missing a sc bristle	6%	0%	0%	0%	5%	4%
		Missing p sc bristle	0%	0%	0%	0%	0%	0%

[1]Percentages that are in **red** reflect a decrease in penetrance of ≥10% compared to the controls of the relevant sex; percentages that are in **green** reflect an increase in penetrance of ≥10% compared to the controls of the relevant sex.
[2]Although the penetrance of the rough and reduced eye phenotype was 100% for all genotypes and sexes, the phenotype was enhanced in the *Marcal1* loss-of-function and overexpression backgrounds.
[3]Only hemizygous males were assessed since the phenotype of interest is only present in homozygous females and hemizygous males and the *Marcal1* overexpression cross did not give rise to homozygous female progeny.
[4]Since several features of the Delta mutant phenotype and the *Marcal1* overexpression phenotype overlap, it was not possible to assess these wings. However, as evidence of a genetic interaction between *Dl* and *Marcal1*, a proportion of flies presented with the new phenotype of blistered wings. Percentages of blistered wings observed are shown.

Abbreviations: ACV, anterior crossvein; a dc, anterior dorsocentral; a np; anterior notopleural; a pa; anterior postalar; a sa, anterior supraalar; a sc, anterior scutellar; L, longitudinal vein; l h, lower humeral; NA, not applicable; PCV, posterior crossvein; p dc, posterior dorsocentral; p np; posterior notopleural; p pa, posterior postalar; p s, presutural; p sa, posterior supraalar; p sc, posterior scutellar; u h, upper humeral.

SUPPLEMENTARY FIGURES

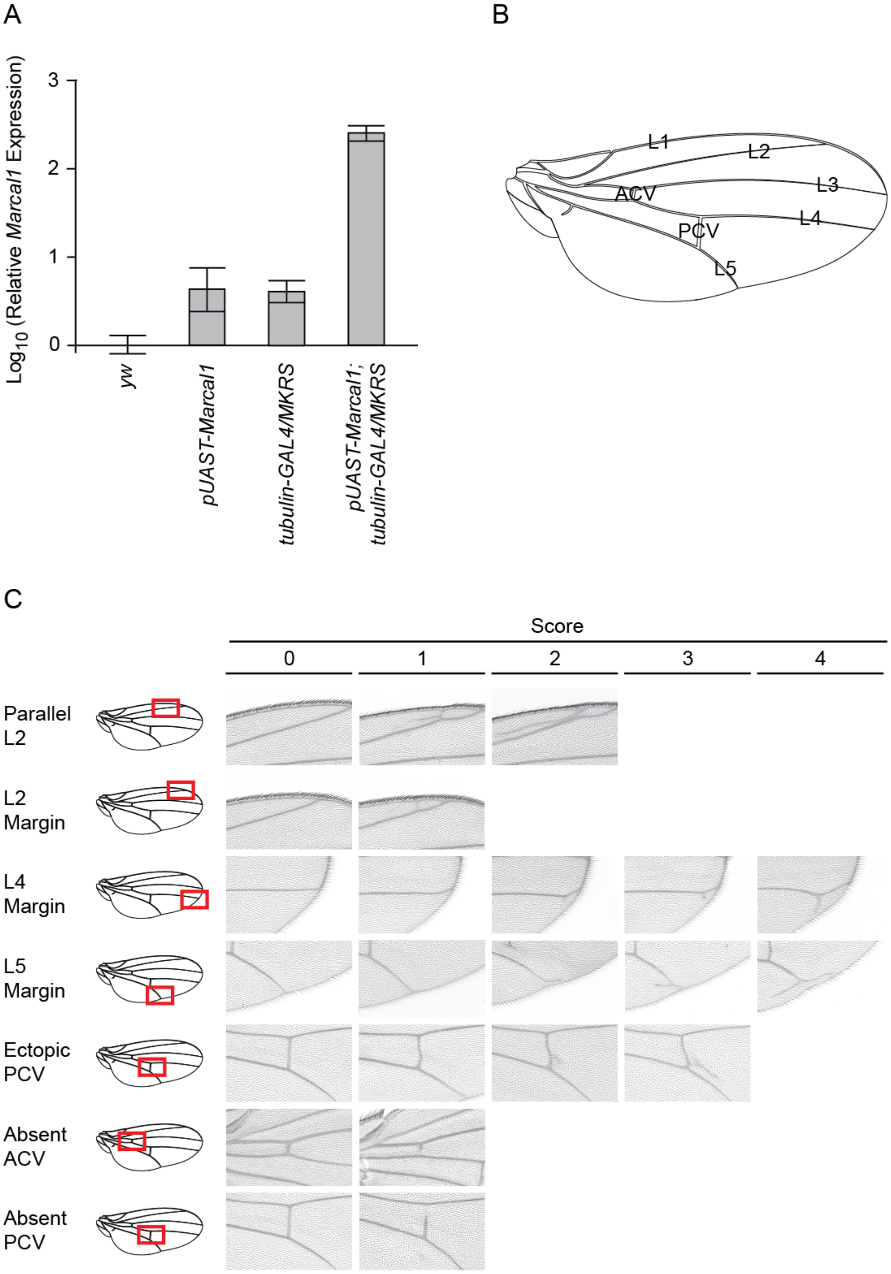

Figure S1. Gene expression analysis of *Marcal1* mRNA in the *Marcal1* overexpression transgenic line and the scoring reference used for scoring ectopic wing veins in the genetic screen. (A) Relative *Marcal1* mRNA expression measured by quantitative PCR in *yw*; *pUAST-Marcal1*; *tubulin-GAL4/MKRS*; and *pUAST-Marcal1, tubulin-GAL4/MKRS* adult flies. The *Marcal1* mRNA levels of three technical replicates were normalized to the mRNA levels of the housekeeping gene *Gapdh2* and the mean *Marcal1* mRNA level was plotted relative to *yw*. Error bars represent one standard deviation. (B) A representation of a wild type *Drosophila* wing showing the 5 longitudinal veins (L1-L5) and the 2 crossveins (ACV and PCV). (C) The scoring reference showing the region of the wing analyzed (red box) and the representative phenotypes for each feature and the corresponding score that would be assessed. An ectopic vein parallel to the L2 vein; veins at the L2, L4, and L5 wing margins; an ectopic vein perpendicular to the PCV; and the absence of the ACV and PCV were assessed. Abbreviations: ACV, anterior cross vein; L, longitudinal; PCV, posterior cross vein.

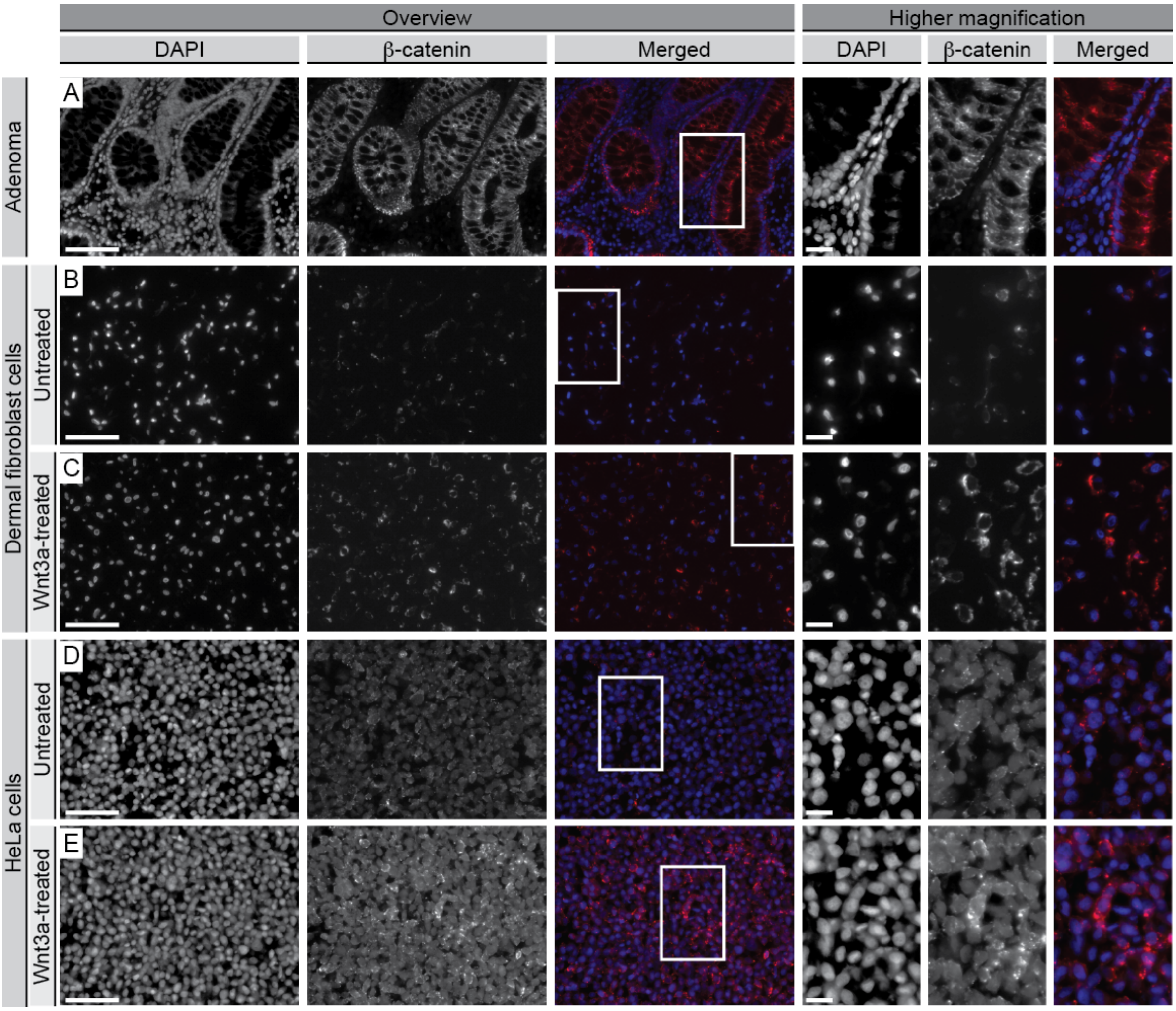

Figure S2. Positive controls for the unphosphorylated β-catenin immunofluorescent staining. Immunostaining with anti-unphosphorylated β-catenin (Alexa 594) of an adenoma from a familial adenomatous polyposis patient (A), untreated and Wnt3a-treated dermal fibroblast cells (B and C, respectively), and untreated and Wnt3a-treated HeLa cells (D and E, respectively). The nuclei were counterstained with 4′, 6-diamidino-2-phenylindole (DAPI). The boxed regions correspond to the higher magnification images. Scale bars: overview images (200×) = 100 microns; higher magnification images (400×) = 100 microns. Abbreviation: DAPI, 4′, 6-diamidino-2-phenylindole.

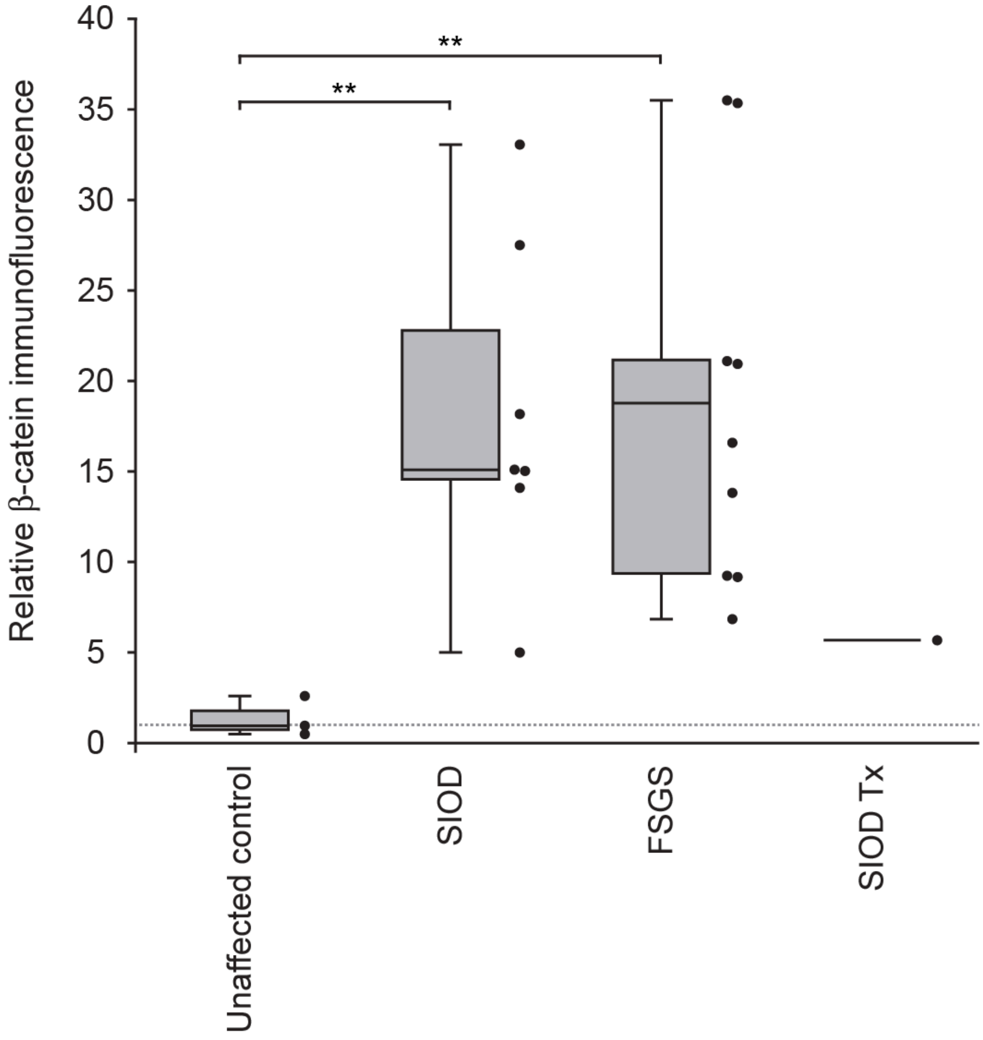

Figure S3. Quantification of β-catenin immunofluorescent staining in SIOD and isolated FSGS kidneys. Expression data are presented as box and whisker plots of relative β-catenin immunofluorescence in unaffected control (n = 3), SIOD patient (n = 7), and isolated FSGS patient (n = 9) kidneys. A single transplanted kidney in an SIOD patient was also available for analysis (SIOD Tx). Boxes represent the interquartile range (25th - 75th percentile), horizontal lines within boxes represent the median, and whiskers represent the range. Adjacent to the box and whisker plots are the individual data points from which the box and whisker plots are derived. β-catenin immunofluorescence values were normalized to the median unaffected control value set at 1 (dotted line). Abbreviations: **, $p < 0.01$; FSGS, focal segmental glomerulosclerosis; SIOD, Schimke immuno-osseous dysplasia.

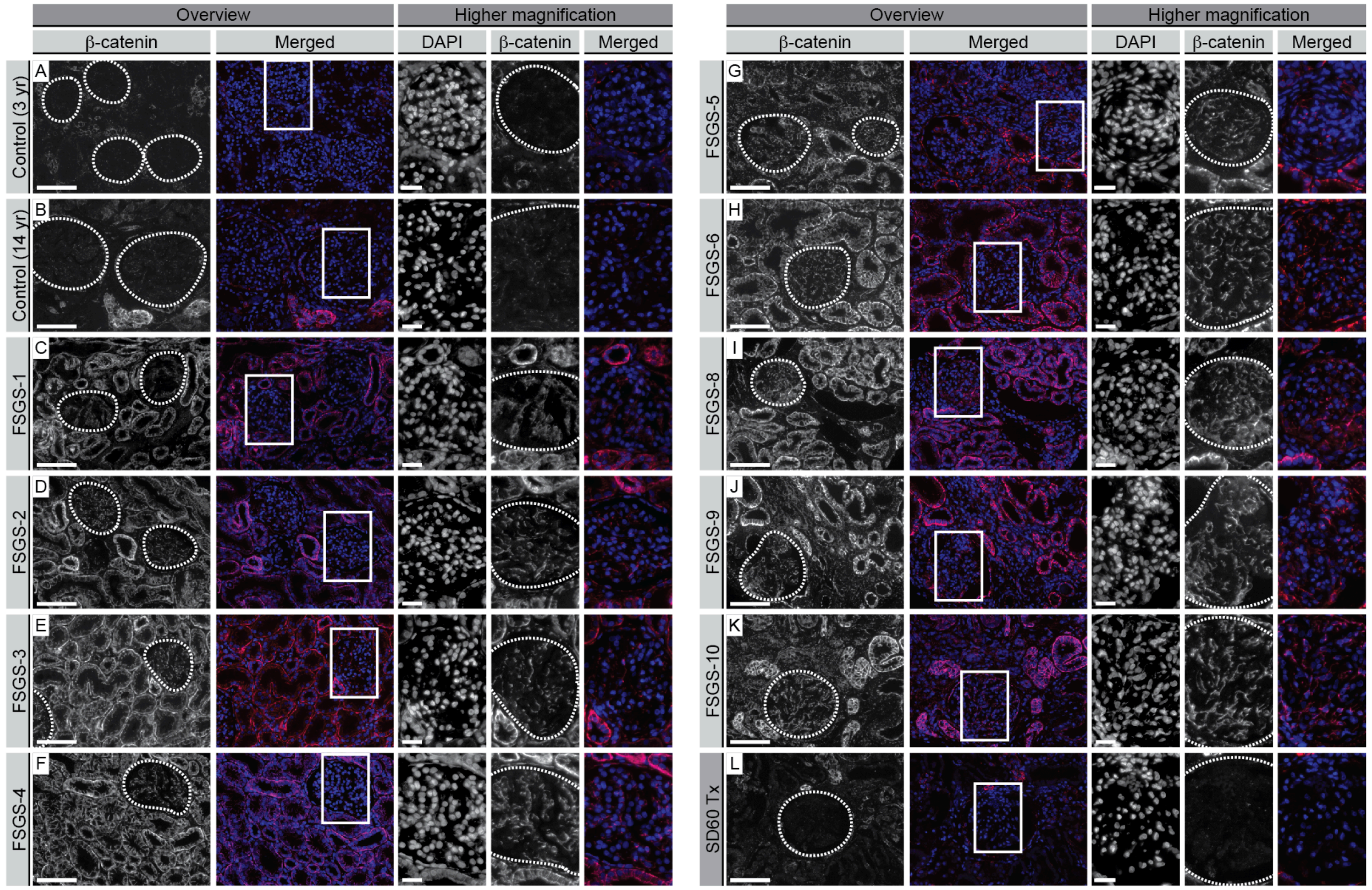

Figure S4. Photomicrographs showing the immunofluorescent detection of the expression and localization of unphosphorylated β-catenin in the glomerular cells of additional unaffected control kidneys, isolated FSGS patient kidneys, and a transplanted kidney in an SIOD patient. Immunostaining with anti-unphosphorylated β-catenin (Alexa Fluor 594) in unaffected control kidneys (A and B), isolated FSGS patient kidneys (C-K), and a transplanted kidney in an SIOD patient (L). The nuclei were counterstained with 4′, 6-diamidino-2-phenylindole (DAPI). The boxed regions correspond to the higher magnification images on the right. The glomeruli have been outlined to aid in the visualization of β-catenin expression. These are representative glomeruli. Scale bars: overview images (200×) and higher magnification images (400×) = 100 microns. Abbreviations: DAPI, 4′, 6-diamidino-2-phenylindole; FSGS, focal segmental glomerulosclerosis; SIOD, Schimke immuno-osseous dysplasia; Tx, transplant; yr, year.

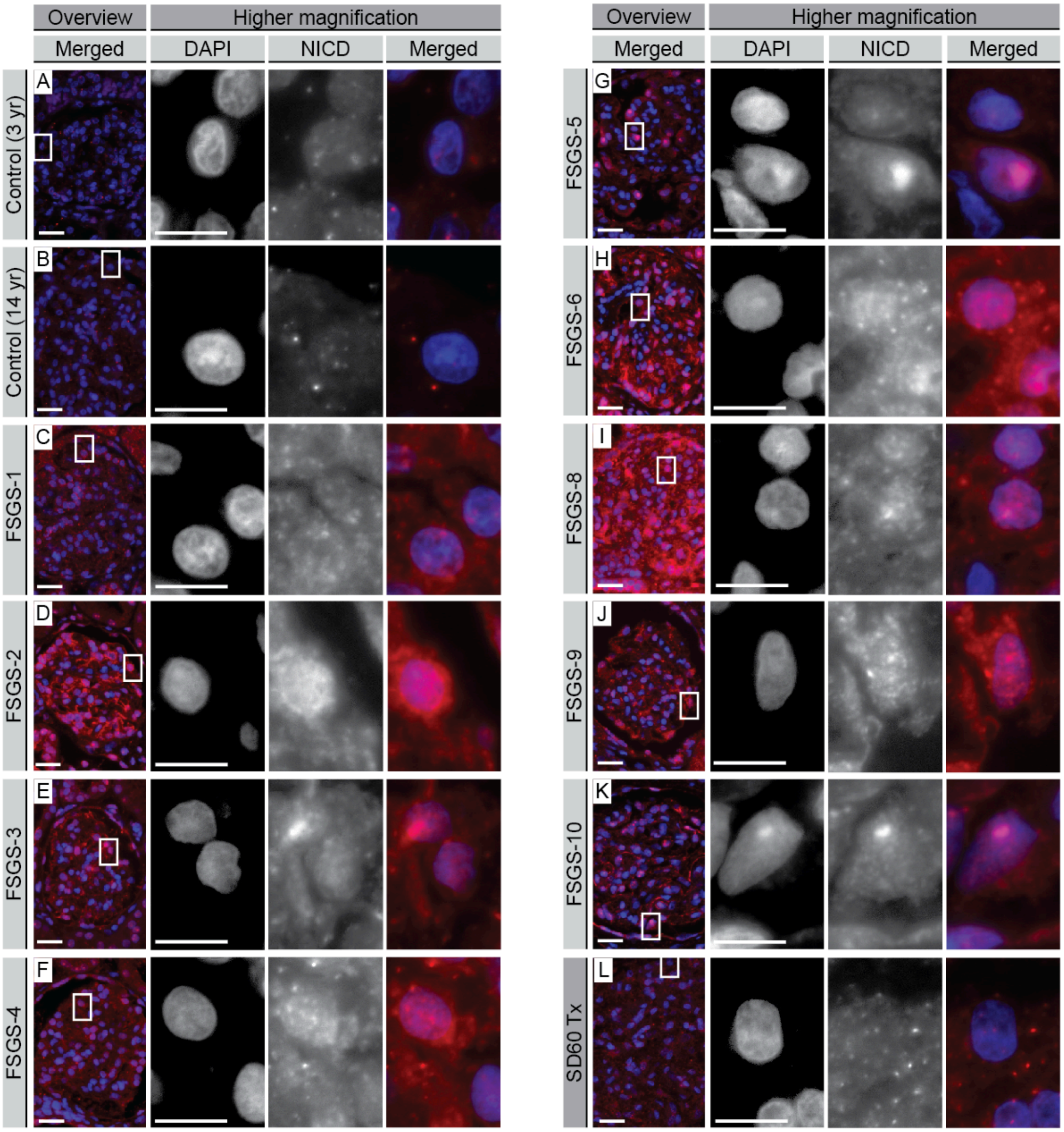

Figure S5. Photomicrographs showing the immunofluorescent detection of the expression and localization of the Notch1 intracellular domain (NICD) in the glomerular cells of additional unaffected control kidneys, isolated FSGS patient kidneys, and a transplanted kidney in an SIOD patient. Immunostaining with anti-NICD (Alexa Fluor 594) in unaffected control kidneys (A and B), isolated FSGS patient kidneys (C-K), and a transplanted kidney in an SIOD patient (L). The nuclei were counterstained with 4′, 6-diamidino-2-phenylindole (DAPI). The boxed regions on the left correspond to the higher magnification images on the right. These are representative glomeruli. Scale bars: overview images (400×) = 100 microns; higher magnification images (1000×) = 10 microns. Abbreviations: DAPI, 4′, 6-diamidino-2-phenylindole; FSGS, focal segmental glomerulosclerosis; NICD, Notch1 intracellular domain; SIOD, Schimke immuno-osseous dysplasia; yr, year.

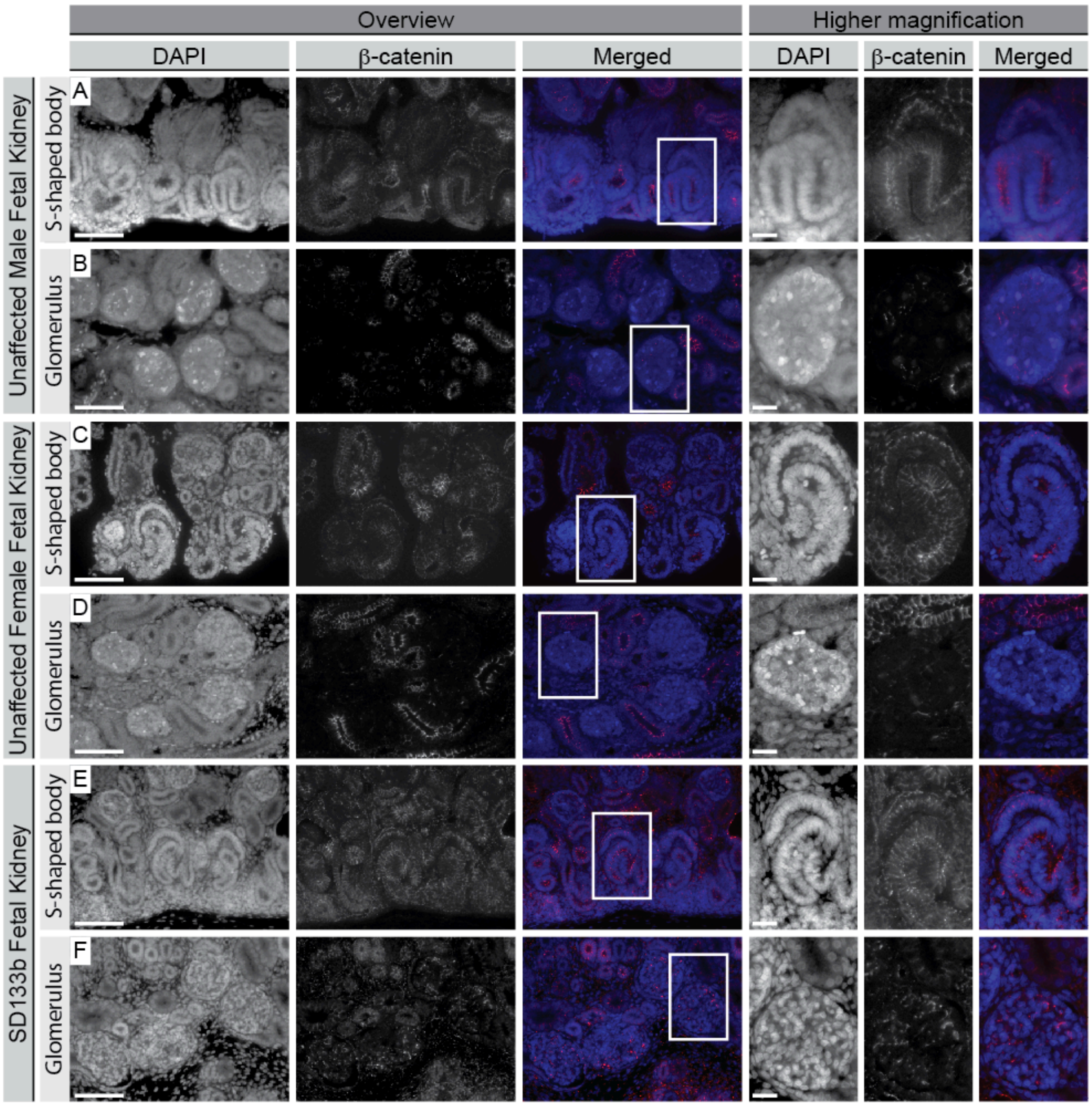

Figure S6. Photomicrographs showing the immunofluorescent detection of the expression and localization of unphosphorylated β-catenin in the 15-week-gestation SMARCAL1-deficient and unaffected control fetal kidneys. Immunostaining with anti-unphosphorylated β-catenin (Alexa 594) in the S-shaped bodies and glomeruli of a 15-week-gestation unaffected male (A and B, respectively) and female (C and D, respectively) and of a 15-week-gestation SMARCAL1-deficient female (E and F, respectively). The nuclei were counterstained with 4′, 6-diamidino-2-phenylindole (DAPI). The boxed regions correspond to the higher magnification images. These are representative S-shaped bodies and glomeruli. Scale bars: overview images (200×) = 100 microns; higher magnification images (400×) = 100 microns. Abbreviation: DAPI, 4′, 6-diamidino-2-phenylindole.

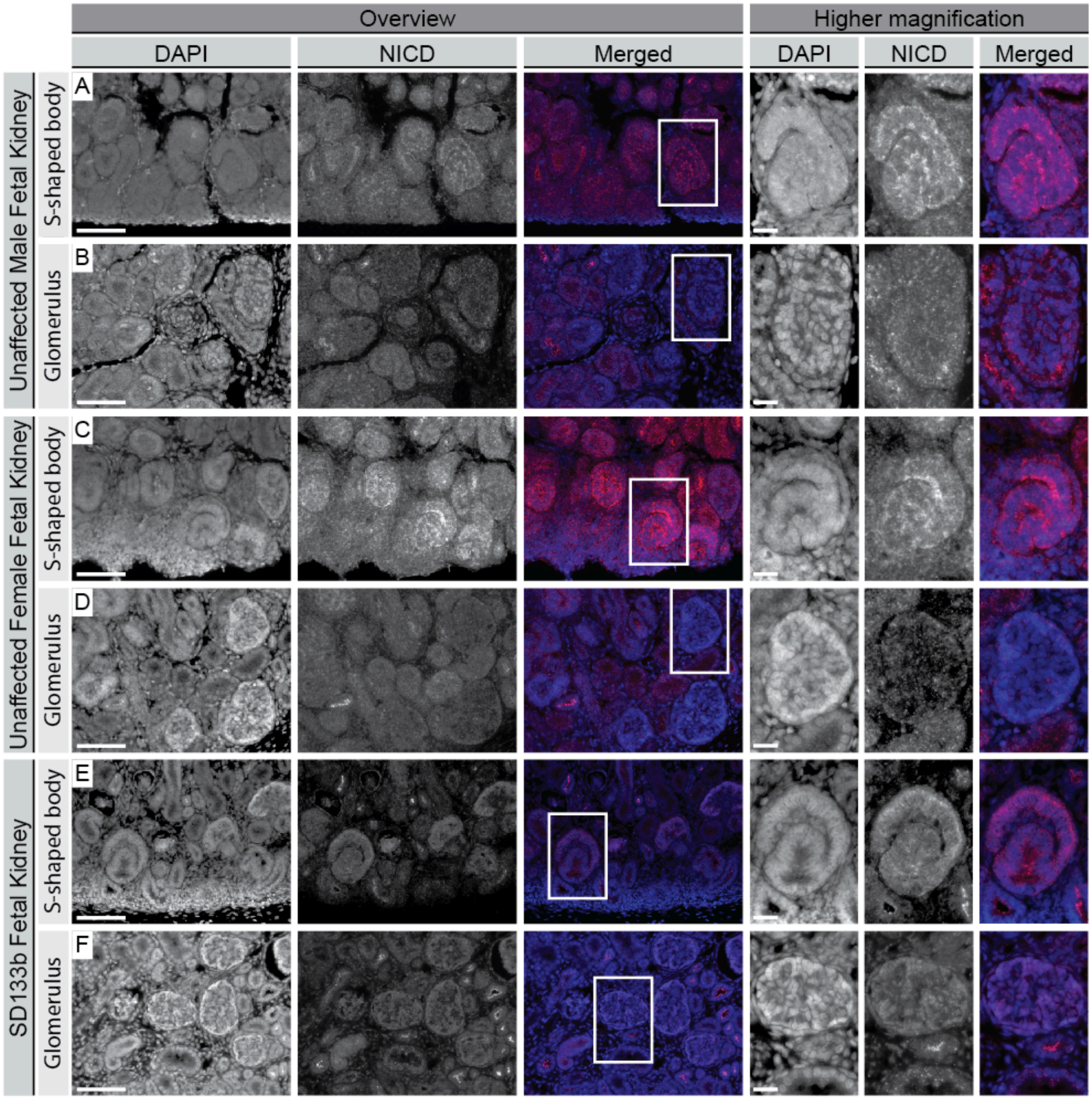

Figure S7. Photomicrographs showing the immunofluorescent detection of the expression and localization of the Notch1 intracellular domain (NICD) in the 15-week-gestation SMARCAL1-deficient and unaffected control fetal kidneys. Immunostaining with anti-NICD (Alexa Fluor 594) in the S-shaped bodies and glomeruli of a 15-week-gestation unaffected male (A and B, respectively) and female (C and D, respectively) and of a 15-week-gestation SMARCAL1-deficient female (E and F, respectively). The nuclei were counterstained with 4′, 6-diamidino-2-phenylindole (DAPI). The boxed regions correspond to the higher magnification images. These are representative S-shaped bodies and glomeruli. Scale bars: overview images (200×) = 100 microns; higher magnification images (400×) = 100 microns. Abbreviations: DAPI, 4′, 6-diamidino-2-phenylindole; NICD; Notch1 intracellular domain.

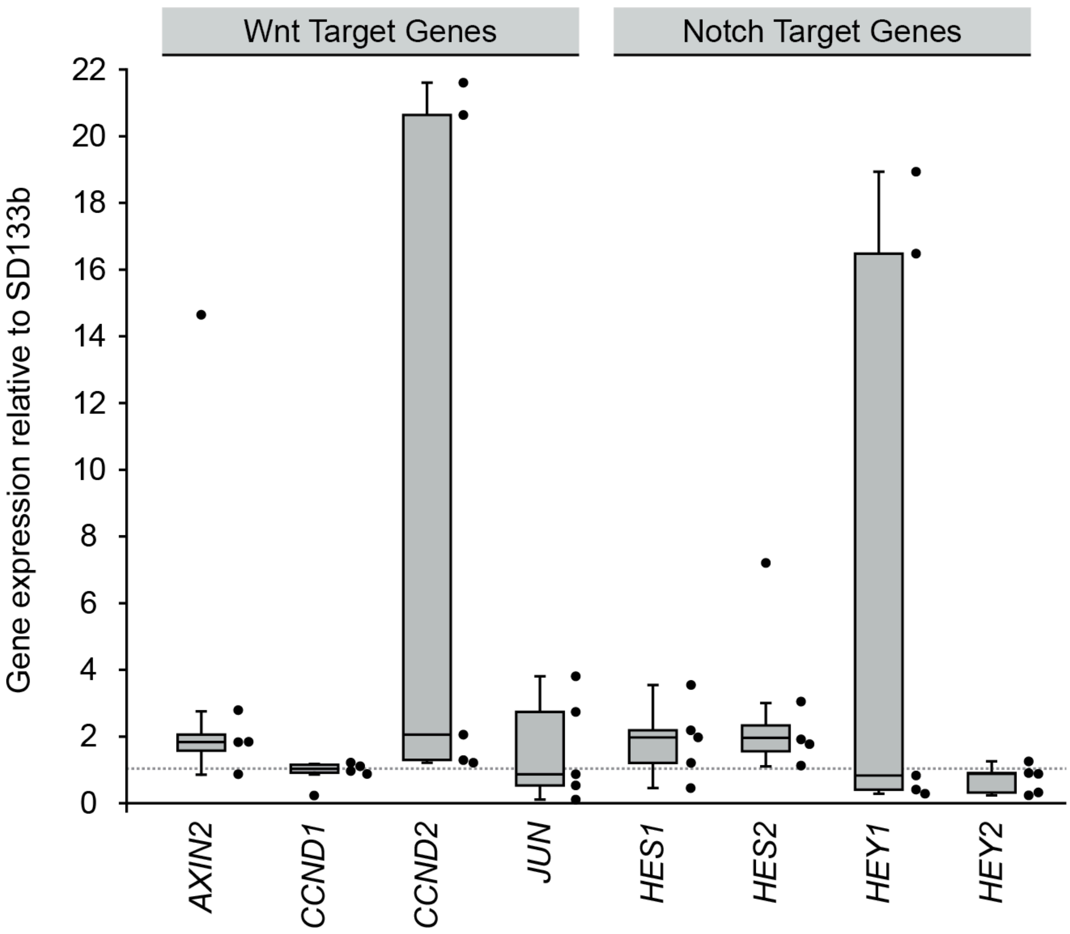

Figure S8. Graph showing the relative expression of target genes of the Wnt and Notch signaling pathways in the 15-week-gestation unaffected versus SMARCAL1-deficient fetal kidney. Relative expression of Wnt pathway target genes (*AXIN2*, *CCND1*, *CCND2*, and *CTNNB1*) and Notch pathway target genes (*HES1*, *HES2*, *HEY1*, and *HEY2*) were measured by qRT-PCR in 15-week-gestation unaffected control kidneys (n = 4) and in a 15-week-gestation SMARCAL1-deficient kidney (n = 1, SD133b). For each sample, the mRNA levels of 3 technical replicates were normalized to the mRNA levels of the housekeeping gene *GAPDH* and plotted relative to SD133b set at 1 (dotted line). Boxes represent the interquartile range (25th - 75th percentile), horizontal lines within boxes represent the median, whiskers represent the range, and individual points represent outliers. Adjacent to the box and whisker plots are the individual data points from which the box and whisker plots are derived.

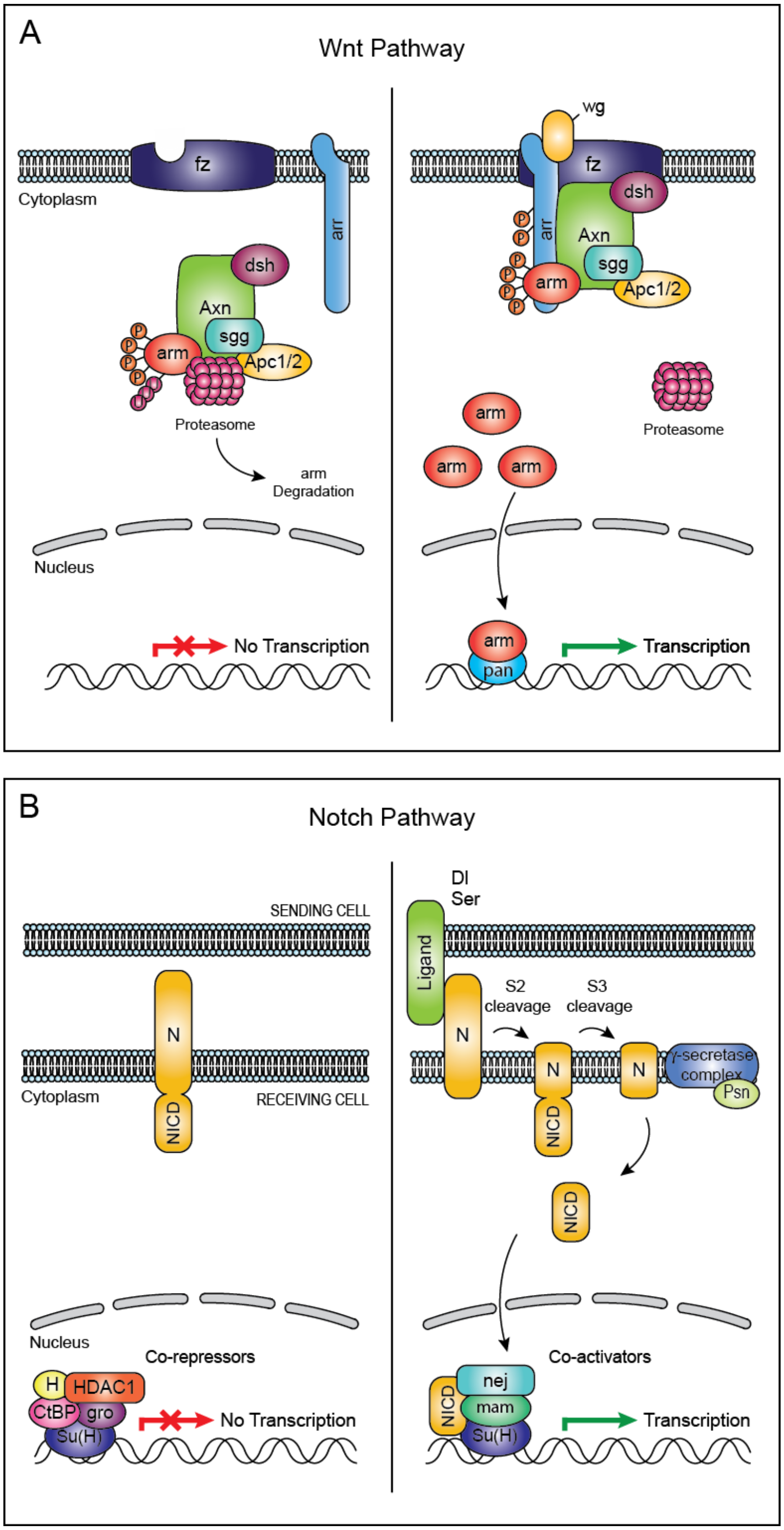
A
Wnt Pathway
fz
Cytoplasm
arr
dsh
Axn
sgg
arm
Apc1/2
Proteasome
arm
Degradation
Nucleus
No Transcription
wg
fz
dsh
arr
Axn
sgg
arm
Apc1/2
arm
arm
arm
Proteasome
arm
pan
Transcription
B
Notch Pathway
SENDING CELL
N
Cytoplasm
RECEIVING CELL
NICD
Nucleus
Co-repressors
H
HDAC1
CtBP
gro
Su(H)
No Transcription
Dl
Ser
Ligand
S2
cleavage
S3
cleavage
N
N
N
γ-secretase
complex
Psn
NICD
NICD
Co-activators
nej
NICD
mam
Su(H)
Transcription

Figure S9. A schematic of the *Drosophila* Wnt and Notch signaling pathways. (A) Wnt pathway schematic: In the absence of the wingless (wg) ligand, armadillo (arm) is phosphorylated by shaggy (sgg), ubiquinated, and targeted for degradation via the proteasome. In the presence of wg, the complex associates with phosphorylated arrow (arr). Although arm is still phosphorylated by sgg, ubiquitination of arm is inhibited. The complex becomes saturated with phosphorylated arm, and newly synthesized arm accumulates and translocates to the nucleus to activate target gene expression. (B) Notch pathway schematic: In the absence of ligand, the Notch pathway is inactive. Suppressor of hairless (Su(H)) binds Hairless (H) to recruit co-repressors such as C-terminal binding protein (CtBP), groucho (gro), and histone deacetylase (HDAC1) to repress transcription. In the presence of the ligand Delta (Dl) or Serrate (Ser), the ligand binds Notch (N) to trigger consecutive S2 and S3 proteolytic cleavages of N; Presenilin (Psn) is part of the γ-secretase complex which is involved in the S3 cleavage. The Notch intracellular domain (NICD) is then released into the cytoplasm and translocates to the nucleus where it binds Su(H) and recruits the co-activators such as mastermind (mam) and nejire (nej) to activate transcription.

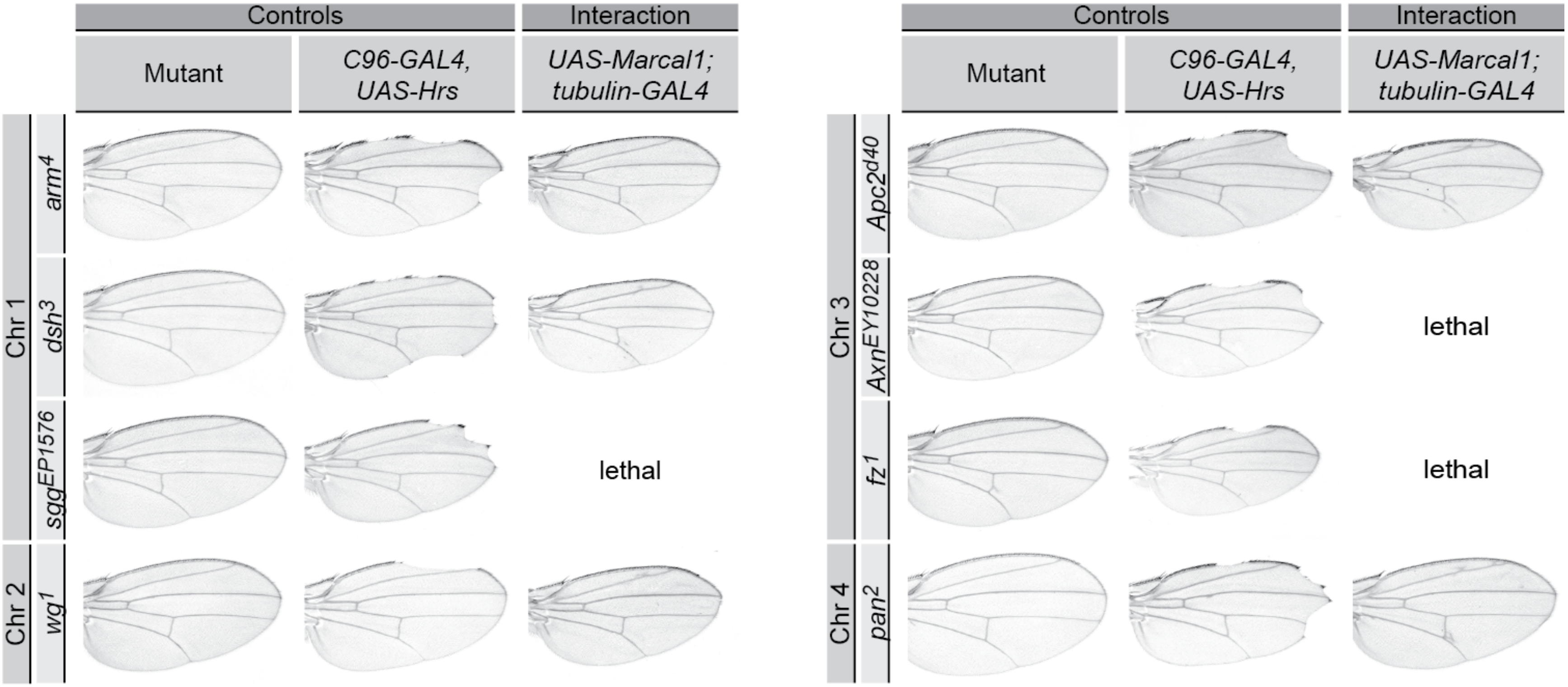

Figure S10. Genetic interaction of *Marcal1* with the Wnt signaling pathway mutants. Representative F_1 wings of crosses between the *Marcal1* overexpression line and the Wnt pathway mutants are shown on the right. Representative wings of each of the Wnt pathway mutants are shown on the left. Representative F_1 wings of crosses between the *C96-GAL4 UAS-Hrs/MKRS* line and the Wnt pathway mutants are shown in the centre. The mutant alleles and chromosomal location of the gene of interest are shown. All wings shown are from female flies. Abbreviations: Chr, chromosome; UAS, upstream activating sequence.

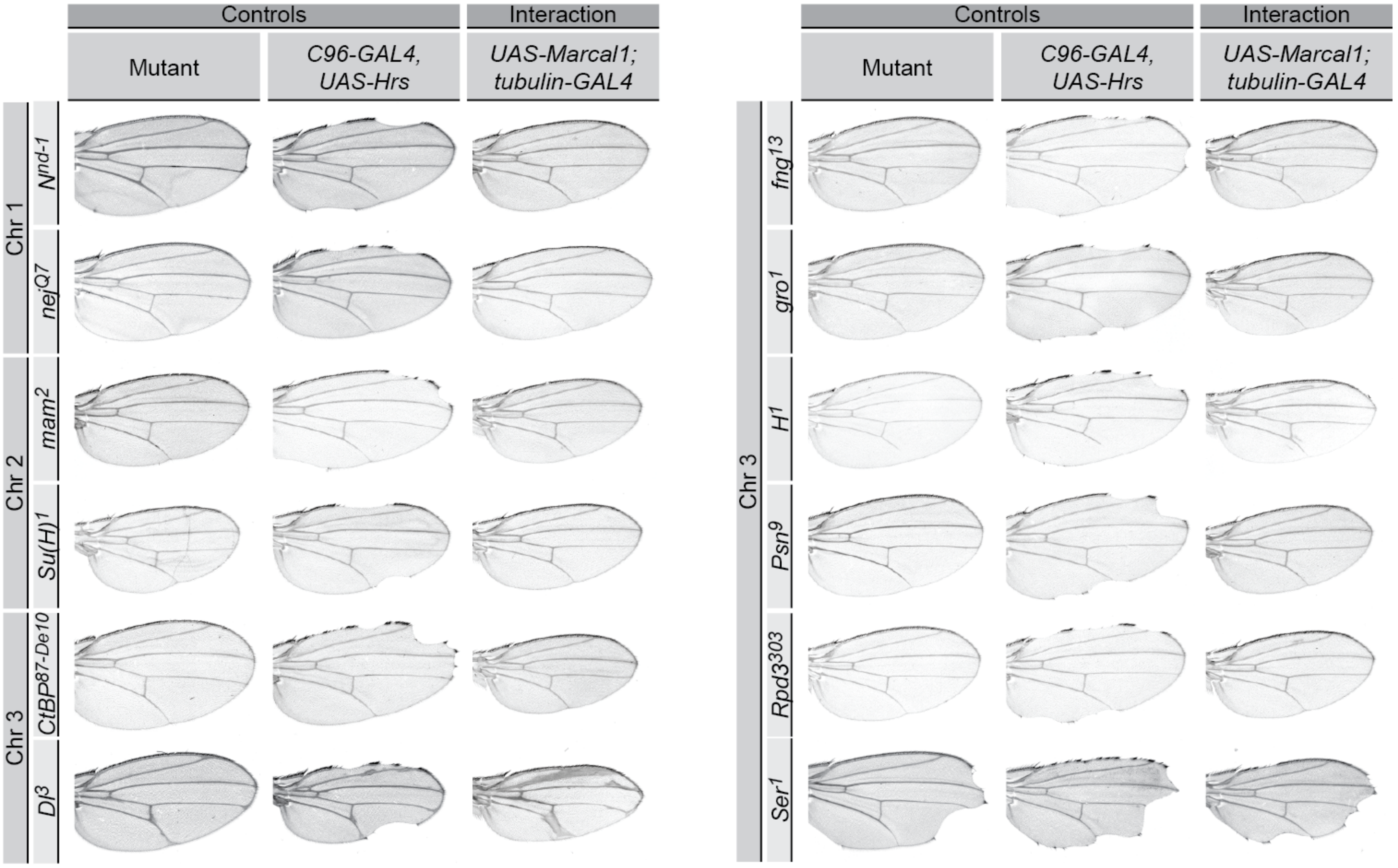

Figure S11. Genetic interaction of *Marcal1* with the Notch signaling pathway mutants. Representative F_1 wings of crosses between the *Marcal1* overexpression line and the Notch pathway mutants are shown on the right. Representative wings of each of the Notch pathway mutants are shown on the left. Representative F_1 wings of crosses between the *C96-GAL4 UAS-Hrs/MKRS* line and the Notch pathway mutants are shown in the center. The mutant alleles and chromosomal location of the gene of interest are shown. All wings shown are from female flies. Abbreviations: Chr, chromosome; UAS, upstream activating sequence.

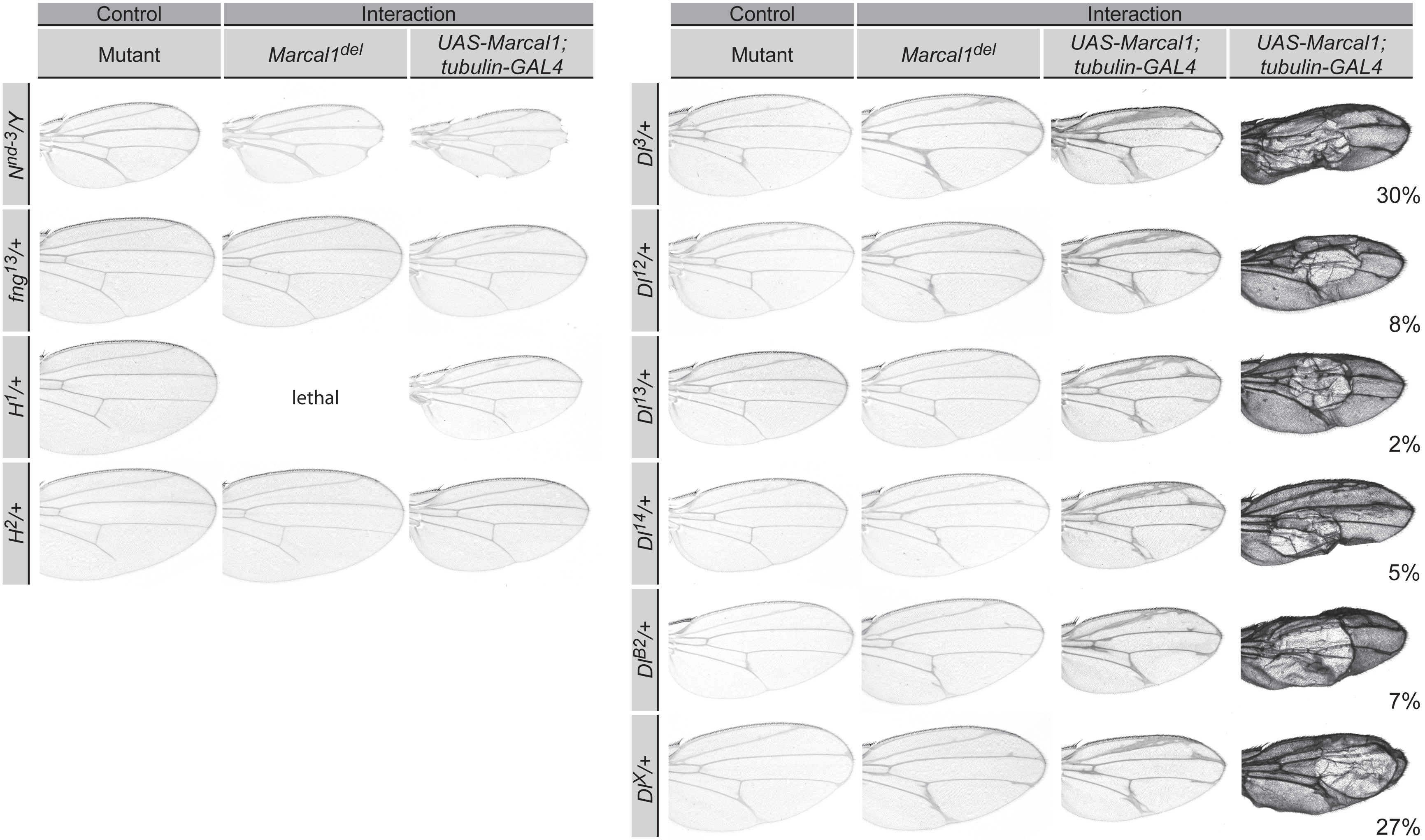

Figure S12. Genetic interaction of *Marcal1* loss and gain with Notch signaling pathway mutant alleles. Representative wings of the mutant of interest (left columns), the mutant allele in the *Marcal1* loss-of-function background (middle and middle left columns), and the mutant allele in the *Marcal1* overexpression background (right and middle right columns). Since several features of the Delta mutant phenotype and the *Marcal1* overexpression phenotype overlap, it was not possible to assess these wings (middle right column), however a proportion of flies presented with the new phenotype of blistered wings (right column), indicative of a genetic interaction between *Marcal1* gain and Delta. Percentages of blistered wings observed are shown. All wings shown are from female flies, excepting the N^{nd-1} allele since the *N* phenotype of interest is only present in homozygous females and hemizygous males and the *Marcal1* overexpression cross did not give rise to homozygous female progeny.

REFERENCES

1. Baradaran-Heravi A, Cho KS, Tolhuis B, Sanyal M, Morozova O, Morimoto M, et al. Penetrance of biallelic SMARCAL1 mutations is associated with environmental and genetic disturbances of gene expression. Hum Mol Genet. 2012;21:2572-87.
2. Pepple KL, Anderson AE, Frankfort BJ, Mardon G. A genetic screen in Drosophila for genes interacting with senseless during neuronal development identifies the importin moleskin. Genetics. 2007;175:125-41.
3. Lindsley DL, Zimm GG. The Genome of Drosophila melanogaster. San Diego, California: Academic Press; 1992.
4. Belt AL. A non-lethal allele of Serrate? Drosoph Inf Serv. 1971;46:116.
5. Bang AG, Hartenstein V, Posakony JW. Hairless is required for the development of adult sensory organ precursor cells in Drosophila. Development. 1991;111:89-104.
6. Kahali B, Bose A, Karandikar U, Bishop CP, Bidwai AP. On the mechanism underlying the divergent retinal and bristle defects of M8* (E(spl)D) in Drosophila. Genesis. 2009;47:456-68.
7. Knust E, Bremer KA, Vassin H, Ziemer A, Tepass U, Campos-Ortega JA. The enhancer of split locus and neurogenesis in Drosophila melanogaster. Dev Biol. 1987;122:262-73.
